# Fast approximate inference for multivariate longitudinal data

**DOI:** 10.1093/biostatistics/kxab021

**Published:** 2021-05-15

**Authors:** David M Hughes, Marta García-Fiñana, Matt P Wand

**Affiliations:** Department of Health Data Science, Waterhouse Building, Block F, University of Liverpool, 1-5 Brownlow Street, Liverpool, L69 3GL, UK; Department of Health Data Science, Waterhouse Building, Block F, University of Liverpool, 1-5 Brownlow Street, Liverpool, L69 3GL, UK; School of Mathematical and Physical Sciences, University of Technology Sydney, P.O. Box 123, Broadway, NSW 2007, AUSTRALIA

**Keywords:** Bayesian computing, Generalized linear mixed model, Markov chain Monte Carlo, Mean field variational Bayes, Multivariate mixed models, Repeated measurements

## Abstract

Collecting information on multiple longitudinal outcomes is increasingly common in many clinical settings. In many cases, it is desirable to model these outcomes jointly. However, in large data sets, with many outcomes, computational burden often prevents the simultaneous modeling of multiple outcomes within a single model. We develop a mean field variational Bayes algorithm, to jointly model multiple Gaussian, Poisson, or binary longitudinal markers within a multivariate generalized linear mixed model. Through simulation studies and clinical applications (in the fields of sight threatening diabetic retinopathy and primary biliary cirrhosis), we demonstrate substantial computational savings of our approximate approach when compared to a standard Markov Chain Monte Carlo, while maintaining good levels of accuracy of model parameters.

## 1. Introduction

Since the random-effects models paper of Laird and Ware (1982), mixed models have become a standard tool for the analysis of longitudinal data in medical studies. The aim is to capture the evolution over time of a marker of interest. Fixed effects describe the influence of covariates on the population mean profile, and random effects describe the group-specific deviations from the population mean.

Mixed models are now so well established that a number of books could introduce the reader to the basic principles and many extensions of methods for analyzing longitudinal data. See for example Verbeke and Molenberghs (2000) for details of the linear mixed model, mainly focusing on continuous longitudinal outcomes, or Molenberghs and Verbeke (2005), McCulloch *and others* (2008) or Diggle *and others* (2002) for further details of various extensions including modeling noncontinuous responses with generalized linear mixed models.

Our focus is on the longitudinal analysis of medical data, with measurements of multiple clinical variables collected repeatedly over time. However, at its most basic level, our problem is the analysis of *grouped* data. Such data are commonly found in a wide range of applications, not limited to medical data, including *panel data analysis* (Baltagi, 2008), *multilevel models* (Gelman and Hill, 2007; Goldstein, 2011), and *small area estimation* (Rao and Molina, 2015).

Extensions have been developed in many directions. We consider the case where multiple longitudinal outcomes are observed on each patient in a clinical study. In such studies, both repeated observations of a marker on the same patient, and observations of different markers on the same patient are likely to be correlated, and modeling strategies should account for the correlation implied by this hierarchical structure. For example, patients with diabetes will routinely have their glycated hemoglobin (HbA1c), cholesterol, blood pressure, and estimated glomerular filtration rate (eGFR) measured (along with potentially many other outcomes, including level of retinopathy collect at retinal screening visits for example), to allow monitoring of a patient’s diabetes severity and assessment of the risk of developing additional complications such as sight threatening diabetic retinopathy.

Many studies that collect longitudinal data on multiple outcomes analyze longitudinal trends separately using univariate mixed models. Depending on the questions of interest this may be a legitimate approach. However, by analyzing outcomes separately we are unable to describe the relationship between outcomes or to assess the simultaneous effect of some covariate on a number of related outcomes. To answer these questions, multivariate mixed models can be used.

Verbeke *and others* (2014) provide a review of various ways of analyzing multivariate longitudinal data. Our work in this article concerns the conditional models in Section 3 of their review. Constructing multivariate mixed models for longitudinal data involves a trade-off between the information gained in such models and the computational cost of fitting the model. For this reason, most work on multivariate mixed models only considers the inclusion of a small number of longitudinal markers (typically 2–5 markers). A notable exception is a pairwise approach that considers all combinations of bivariate longitudinal models to assess changes over time in 22 hearing threshold frequencies (Fieuws *and others*, 2007). A key problem is that the inclusion of more longitudinal markers, usually also involves the specification of higher-dimensional random effects distributions. This makes maximum likelihood estimation challenging due to the need to evaluate high-dimensional integrals over random effects distributions. Bayesian estimation through Markov Chain Monte Carlo (MCMC) can also be computationally challenging due to the high-dimensional nature of the problem.

In this article, we propose mean field variational Bayes (MFVB) as an efficient way of fitting multivariate mixed models. MFVB is widely used in computer science (e.g. Bishop, 2006) though is perhaps less familiar in the statistical literature. This situation is changing, thanks in part to two recent reviews of MFVB (Blei *and others*, 2017) and the related variational message passing (Wand, 2017).

In Section 2, we describe multivariate generalized linear mixed models (MGLMMs) and provide a Bayesian specification of the model of interest in this paper. Section 3 gives a brief overview of MFVB methods and describes in detail the computations necessary to develop an algorithm for MFVB estimation of MGLMMs. We assess the performance of our MFVB algorithm in comparison to popular MCMC routines, in simulated data sets in Section 4, whilst in Section 5, we show the performance of our approach in a relatively small data set of patients with primary biliary cirrhosis and a much larger dataset of patients with diabetes who were screened for sight threatening diabetic retinopathy. Section 6 provides a brief conclusion to the article.

## 2. Multivariate generalized linear mixed models

### 2.1. Notation

We begin by first describing the notation used in this article. We consider a study that collects data on $m$ individuals. For each patient, data are collected on up to $R$ longitudinal markers of interest. We let $y_{irj}$ denote the $j$th observation ($j=1,\ldots,n_{ir}$) of the $r$th marker ($r=1,\ldots,R$) for patient $i$, ($i=1,\ldots,m$) and collect all observations of marker $r$ on a particular patient into a vector $\mathbf{y}_{ir}=(y_{ir1},\ldots,y_{irn_{ir}})^T$. Further, we collect all observations of the $R$ markers on a particular patient into a combined vector $\mathbf{y}_i = (\mathbf{y}_{i1}^T,\ldots,\mathbf{y}_{iR}^T)^T$, and let $\mathbf{y}=(\mathbf{y}_1^T,\ldots,\mathbf{y}_m^T)^T$ denote all the longitudinal observations for the study in question. We may specify some covariates that are believed to influence the change over time in each longitudinal marker. The covariates for each marker, $r$, for each individual, $i$, are stored in a ($n_{ir} \times p_r$) design matrix $\mathbf{X}_{ir}$. The overall design matrix for individual $i$ is represented by $\mathbf{X}_i = \text{blockdiag}(\mathbf{X}_{i1},\ldots,\mathbf{X}_{iR})$. Similar design matrices can be constructed for the random effects terms in a mixed model, which are denoted by the $(n_i \times q)$ matrix $\mathbf{Z}_i = \text{blockdiag}(\mathbf{Z}_{i1},\ldots,\mathbf{Z}_{iR})$, where $q=\sum_{r=1}^R q_r$ denotes the total number of random effects included in the model, and $n_i=\sum_{r=1}^Rn_{ir}$ denotes the total number of measurements on individual $i$. These design matrices can be stacked across all individuals, giving $\mathbf{X} = [\mathbf{X}_1^T,\ldots,\mathbf{X}_m^T]^T$ and $\mathbf{Z} = \text{blockdiag}\{\mathbf{Z}_1,\ldots,\mathbf{Z}_m\}$.

### 2.2. Model specification

We now proceed to develop our MGLMM. We assume that each response, $Y_r$ is distributed according to a member of the exponential family, to allow the inclusion of non-continuous responses such as binary and Poisson markers, in addition to continuous markers,

(2.1) \begin{equation*} \mathbf{Y}_r|\mathbf{\beta},\mathbf{u} \sim \exp\{\mathbf{y}_r^T \mathbf{\Sigma}_{\varepsilon_r}^{-1} \mathbf{C_r}{\tilde{\boldsymbol{\nu}}}_r -\mathbf{1}^T\mathbf{\Sigma}_{\varepsilon_r}^{-1} b(\mathbf{C_r}{\tilde{\boldsymbol{\nu}}}_r) -\mathbf{1}^T c(\mathbf{y}_r,\phi_r)\}, \end{equation*}

where for notational convenience we have defined $C=[\mathbf{X} ~ \mathbf{Z}]$ and ${\tilde{\boldsymbol{\nu}}}=(\mathbf{\beta}^T,\mathbf{u}^T)^T$, with $\mathbf{\beta}$ denoting the $p=\sum_{r=1}^R p_r$ fixed effects in the model, and $\mathbf{u}$ denoting the $mq$ vector of individual random effects. The subscript $r$ attached to any of these design matrices denotes the parts relating to marker $r$. We denote by $\mathbf{\Sigma}_{\varepsilon}$ a matrix of nuisance parameters which in our case is a diagonal matrix with diagonal entries of $\sigma^2_{\varepsilon_r}$ if the row corresponds to an observation from the $r$th Gaussian marker, and 1 if the row corresponds to an observation of a binary or count longitudinal marker.

In 2.1, we use $b$ to denote the cumulant function and $c$ to denote the base measure according to the notation of McCullagh and Nelder (1989), and assume elementwise evaluation of the functions $b$ and $c$ (i.e., the appropriate function is applied to the appropriate row of input). For example, if $Y_r$ is a continuous marker $b(x)=x^2/2$ whilst if $Y_r$ is binary then $b(x)= \log(1+e^x)$ and if Poisson, $b(x)=e^x$. To extend this model for our needs in this article to fit a joint model to multiple longitudinal responses, we consider the following density for our stacked response $\mathbf{Y}$,

$$\begin{equation*}
\mathbf{Y}|\mathbf{\beta},\mathbf{u} \sim \exp\{\mathbf{y}^T \mathbf{\Sigma}_{\varepsilon}^{-1} \mathbf{C}{\tilde{\boldsymbol{\nu}}} -\mathbf{1}^T\mathbf{\Sigma}_{\varepsilon}^{-1} b(\mathbf{C}{\tilde{\boldsymbol{\nu}}}) -\mathbf{1}^T c(\mathbf{y},\phi)\}.
\end{equation*}$$

Here, we have abused notation slightly for the sake of neat exposition, and understand $b(x)$ and $c(x)$ to be elementwise application of whichever transformation is appropriate for the type of outcome corresponding to the row in question. We assume that the random effects terms jointly follow a multivariate normal distribution with mean $0$ and unstructured covariance matrix $\mathbf{\Sigma}_R$. That is,

$$\begin{equation*}
\mathbf{u}|\mathbf{\Sigma}_R \sim \text{N}(\bf{0},I_m \otimes \mathbf{\Sigma}_R),
\end{equation*}$$

with $\otimes$ denoting a Kronecker product. The remaining terms in our model are;

(2.2) \begin{align*} \mathbf{\Sigma}_R|a_1,\ldots,a_q & \sim IW(\nu_1+q-1,2\nu \text{diag}\{1/a_1,\ldots,1/a_q\}), &\qquad a_k &\sim IG(1/2, A_k^{-2})\nonumber \\ \sigma^2_{\varepsilon_r} &\sim IG(1/2, a_{\varepsilon_r}^{-1}), \qquad a_{\varepsilon_r} \sim IG(1/2, A_{\varepsilon_r}^{-2}), &\qquad \mathbf{\beta} &\sim N(0,\sigma_{\beta}^2I_p). \end{align*}

In (2.2), we specify an Inverse-Wishart prior for the random effects covariance matrix $\mathbf{\Sigma}_R$ and inverse-gamma priors for the residual variances $\sigma^2_{\varepsilon r}$ for the $R_c$ continuous markers included in the MGLMM. The inclusion of auxiliary variables $a_k$$(k=1,\ldots,q)$, and $a_{\varepsilon_r}$$(r=1,\ldots,R_c)$ follows the extension of Huang and Wand (2013) in order to place weakly informative priors on the covariance terms in $\mathbf{\Sigma}_R$ that are equivalent to the Half-Cauchy distributions proposed by Gelman (2006). The choice of $\nu=2$ allows the standard deviation terms in $\mathbf{\Sigma}_R$ to have Half-t distributions with 2 degrees of freedom, whilst the correlation parameters have uniform distributions over $(-1,1)$. Each auxiliary variable is assumed to independently follow an inverse-gamma distribution as specified in (2.2). A Normal prior with mean $\mathbf{0}$, and variance $\sigma_{\beta}^2I_p$ is placed on the fixed effects parameter $\mathbf{\beta}$.

We desire posterior distributions on $\mathbf{\theta}=(\mathbf{\beta}, \mathbf{u}, \mathbf{\Sigma}_R, a_1,\ldots,a_q, \sigma^2_{\varepsilon_1},\ldots,\sigma^2_{\varepsilon_{R_c}},a_{\varepsilon_1},\ldots, a_{\varepsilon_{R_c}})$. As mentioned in Section 1, one way to proceed would be to use an MCMC sampling routine. However, as we show in Section 5, this can be very computationally intensive in large data sets that contain data on many longitudinal markers. For this reason, in Section 3, we work towards a mean field variational Bayes solution for fitting MGLMMs in high-dimensional data.

## 3. Variational inference

Our aim in Bayesian inference is to find the posterior distribution for the parameters of interest in a model, described by $\mathbf{\theta}$. Mean field variational Bayes aims to provide an approximation to $p(\mathbf{\theta}|y)$ in situations where a full MCMC sampling procedure would be computationally expensive. An introduction to MFVB from a statistical perspective is given in Ormerod and Wand (2010). The basic premise is to approximate the complex posterior $p(\mathbf{\theta}|y)$ (which is challenging to estimate using MCMC), by a simpler density function $q(\mathbf{\theta})$.

### 3.1. Overview of mean field variational Bayes

MFVB achieves substantial computational gains by enforcing a product restriction

$$\begin{equation*}
q(\mathbf{\theta}) = \prod_{i=1}^M q_i(\mathbf{\theta}_i) \qquad \text{for some partition } \{\mathbf{\theta}_1,\ldots,\mathbf{\theta}_M\} \text{ of } \mathbf{\theta}.
\end{equation*}$$

This restriction is known as the *mean field restriction*, hence the optimal solution, $q^*(\mathbf{\theta})$ (from all possible distributions $Q$) is known as the mean field variational Bayes (MFVB) approximation to the actual posterior distribution $p(\mathbf{\theta}|y)$. The challenge of MFVB is to select an optimal $q^*(\mathbf{\theta})$ that is as close as possible, in terms of Kullback–Leibler divergence to the true posterior. To justify this approach, first consider the joint posterior of the parameter vector given the observed data,

$$\begin{equation*}
p(\mathbf{\theta}|\mathbf{y}) =\frac{p(\mathbf{y},\mathbf{\theta})}{p(\mathbf{y})} = \frac{p(\mathbf{y}|\mathbf{\theta})p(\mathbf{\theta})}{p(\mathbf{y})}.
\end{equation*}$$

As explained in Ormerod and Wand (2010), simple algebraic manipulations show that the logarithm of the marginal likelihood satisfies

$$\begin{align*}
\log p(\mathbf{y}) & = \int q(\mathbf{\theta}) \log \left\{\frac{p(\mathbf{y},\mathbf{\theta})}{q(\mathbf{\theta})}\right\}\rm d\mathbf{\theta} + \int q(\mathbf{\theta}) \log \left\{\frac{q(\mathbf{\theta})}{p(\mathbf{\theta}|\mathbf{y})}\right\} \rm d \mathbf{\theta}, \\
& = \log \underline{p}(\mathbf{y}, q) + KL\{q(\mathbf{\theta})||p(\mathbf{\theta}|\mathbf{y})\}.
\end{align*}$$

Since a KL divergence is always non-negative, we have that $p(\mathbf{y}) \geq \underline{p}(\mathbf{y}, q)$, and so minimizing the KL divergence (which is often intractable) is equivalent to maximizing $\underline{p}(\mathbf{y}, q)$ (which is usually more tractable). Hence, we have that

$$\begin{equation*}
q^*(\mathbf{\theta}) = \underset{q\in Q}{\text{arg min}} KL\{q(\mathbf{\theta})||p(\mathbf{\theta}|\mathbf{y})\} = \underset{q\in Q}{\text{arg max}}\underline{p}(\mathbf{y}, q).
\end{equation*}$$

Under the mean field product restriction, the optimal $q$-density functions satisfy

(3.3) \begin{equation*} q_i^*(\mathbf{\theta}_i) \propto \exp \{E_{-\mathbf{\theta}_i}\left[\log p(\mathbf{y},\mathbf{\theta})\right]\} \propto \exp \{E_{-\mathbf{\theta}_i}\left[\log p(\mathbf{\theta}_i|\text{rest})\right]\}, \end{equation*}

with $E_{-\mathbf{\theta}_i}$ denoting the expectation with respect to all parameters in the model except for those in partition $\mathbf{\theta}_i$ (referred to as the *rest*).

MFVB proceeds by determining optimal forms for each partition of $\mathbf{\theta}$ using (3.3), which will result in expression that each depend on other partitions of $\mathbf{\theta}$. These expressions can be iteratively updated until there is negligible increase in $\log \underline{p}(\mathbf{y}, q)$.

### 3.2. Mean field variational Bayes for multiple markers

Having given a sketched outline of the key elements of MFVB approximations, we now develop the MFVB approximation for the MGLMMs described in Section 2. We seek an approximation to the full posterior as follows.

$$\begin{align*}
& p(\mathbf{\beta}, \mathbf{u}, \mathbf{\Sigma}_R, a_1,\ldots,a_q, \sigma^2_{\varepsilon_1},\ldots,\sigma^2_{\varepsilon_{R_c}},a_{\varepsilon_1},\ldots, a_{\varepsilon_{R_c}}|\mathbf{y}), \\
& \approx q(\mathbf{\beta}, \mathbf{u}, \mathbf{\Sigma}_R, a_1,\ldots,a_q, \sigma^2_{\varepsilon_1},\ldots,\sigma^2_{\varepsilon_{R_c}},a_{\varepsilon_1},\ldots, a_{\varepsilon_{R_c}}), \\
& = q(\mathbf{\beta}, \mathbf{u}, a_1,\ldots,a_q, a_{\varepsilon_1},\ldots, a_{\varepsilon_{R_c}})q(\mathbf{\Sigma}_R,\sigma^2_{\varepsilon_1},\ldots,\sigma^2_{\varepsilon_{R_c}}), \\
& = q(\mathbf{\beta}, \mathbf{u}) q(\mathbf{\Sigma}_R) \prod_{r=1}^{R_c}\left\{q(\sigma^2_{\varepsilon_r})q(a_{\varepsilon_r})\right\}\prod_{k=1}^{q}q(a_k).
\end{align*}$$

Note that the second restrictions are induced simply due to assumed independencies in the model specified in Section 2, and place no further restriction on the parameter space. That is, the strength of the MFVB approach depends in this case on the amount of information lost by the approximation by two factors.

Optimal $q$-densities can be calculated according to (3.3). The updates for $q(\sigma^2_{\varepsilon_r})$, $q(a_{\varepsilon_r}),$ and $q(a_k)$ involve only relatively standard calculations and result in optimal densities that are inverse gamma distributions, with arguments according to Algorithm 1. Similarly, $q^*(\mathbf{\Sigma}_R)$ can be shown to be an inverse Wishart distribution. When all of the longitudinal markers are continuous, $q^*(\mathbf{\beta}, \mathbf{u})$ is a multivariate normal distribution. However, when at least some of the longitudinal markers are Poisson or binary, then evaluation of (3.3) no longer leads to a recognizable distribution. This is caused by the need to evaluate $E_q[\exp(\mathbf{C}{\tilde{\boldsymbol{\nu}}})]$ and $E_q[\log(1 + \exp(\mathbf{C}{\tilde{\boldsymbol{\nu}}}))],$ respectively. To overcome this difficulty, we follow the semiparametric MFVB approach outlined by Rohde and Wand (2016) and specify that $q^*(\mathbf{\beta},\mathbf{u},\mathbf{\mu}_{q_{(\mathbf{\beta},\mathbf{u})}},\mathbf{\Sigma}_{q_{(\mathbf{\beta},\mathbf{u})}}) \sim N(\mathbf{\mu}_{q_{(\mathbf{\beta},\mathbf{u})}},\mathbf{\Sigma}_{q_{(\mathbf{\beta},\mathbf{u})}})$. We still need to evaluate the logistic term for binary markers. A number of approaches could be taken to deal with this, either through quadrature, or through the tilted bound of Jaakkola and Jordan (2000). However, we follow the approach of Nolan and Wand (2017) who use Knowles–Minka–Wand updates with Monahan–Stefanski updates to approximate the logistic fragment with a scaled mixture of normal distributions (Knowles and Minka, 2011; Wand, 2014; Monahan and Stefanski, 1989). Full derivations of these optimal $q$-densities are given in the Supplementary material available at *Biostatistics* online.

The updates for $\mathbf{\Sigma}_{q_{(\mathbf{\beta},\mathbf{u})}}$ require calculation of the inverse of a potentially large matrix, which causes a huge computational cost, especially when $m$ is large. However, we can exploit the block-diagonal structure of $\mathbf{\Sigma}_{q_{(\mathbf{\beta},\mathbf{u})}}$ and streamline our MFVB algorithm using the approach of Lee and Wand (2016), in order to substantially improve the computational speed of our algorithm. Further details of the streamlining approach are given in the Supplementary material available at *Biostatistics* online. The full streamlined MFVB approximation for estimating an MGLMM is given in Algorithm 1. As noted by Rohde and Wand (2016) and Nolan and Wand (2017), the semiparametric MFVB algorithm proposed is not guaranteed to converge, although our empirical work suggests this is not a problem most of the time.

Algorithm 1 Streamlined algorithm for multivariate generalized linear mixed models.

1. Initialize $\mathbf{M}_{q(\Sigma_R^{-1})}$ a $q \times q$ positive definite matrix, $\mu_{q_{(1/a_k)}} >0, \, 1\leq k \leq q$, $\mu_{q(1/\sigma^2_{\varepsilon_r})}>0, \, 1\leq r \leq R_c$, and $\mu_{q(1/\sigma^2_{a_r})}>0, \, 1\leq r \leq R_c$;
2. Cycle through updates
3. $\mathbf{S} \leftarrow \mathbf{0}; \mathbf{s} \leftarrow \mathbf{0}$
4. **for**$i = 1,\ldots, m$
5.    $\mathbf{G}_i \leftarrow \mathbf{X}_i^TE[\mathbf{\Sigma}_{\varepsilon_i}^{-1}]\text{diag}\{E_x[b''(\sigma x + \mu)]\}_i \mathbf{Z}_i$;
6.    $\mathbf{H}_i \leftarrow \left\{\mathbf{Z}_i^TE[\mathbf{\Sigma}_{\varepsilon_i}^{-1}]\text{diag}\{E_x[b''(\sigma x + \mu)]\}_i \mathbf{Z}_i + \mathbf{M}_{q(\Sigma_R^{-1})}\right\}$
7.    $\mathbf{S} \leftarrow \mathbf{S} + \mathbf{G}_i\mathbf{H}_i\mathbf{G}_i^T$; $\qquad \mathbf{s} \leftarrow \mathbf{s} + \mathbf{G}_i\mathbf{H}_i \left(\mathbf{M}_{q(\Sigma_R^{-1})}\boldsymbol{\mu}_{q_{(u_i)}} - \mathbf{Z}_i^T(E[\mathbf{\Sigma}_{\varepsilon_i}^{-1}](\mathbf{y}_i-E_x[b'(\sigma x + \mu)]_i))\right)$
8. $\mathbf{\Sigma}_{q(\mathbf{\beta})} \leftarrow \left(\mathbf{X}^TE[\mathbf{\Sigma}_{\varepsilon}^{-1}]\text{diag}\{b''(\sigma x + \mu)\}\mathbf{X} + \sigma_{\beta}^{-2} \mathbf{I}_p - \mathbf{S}\right)^{-1}$
9. $\boldsymbol{\mu}_{q_{(\mathbf{\beta})}} \leftarrow \boldsymbol{\mu}_{q_{(\mathbf{\beta})}} + \mathbf{\Sigma}_{q(\mathbf{\beta})}\left\{\mathbf{X}^TE[\mathbf{\Sigma}_{\varepsilon_i}^{-1}](\mathbf{y}_i-E_x[b'(\sigma x + \mu)] \sigma_{\beta}^{-2}\boldsymbol{\mu}_{q_{(\mathbf{\beta})}}\mathbf{I}_p + \mathbf{s}\right\}$; $\boldsymbol{\mu}_{q_{(\mathbf{\beta})}}^{OLD} \leftarrow \boldsymbol{\mu}_{q_{(\mathbf{\beta})}}$
10. **for**$i = 1,\ldots, m$
11.    $\Sigma_{q(u_i)} \leftarrow \mathbf{H}_i + \mathbf{H}_i\mathbf{G}_i^T\mathbf{\Sigma}_{q(\mathbf{\beta})}\mathbf{G}_i\mathbf{H}_i$
12.    $\boldsymbol{\mu}_{q_{(u_i)}} \leftarrow \boldsymbol{\mu}_{q_{(u_i)}} + \mathbf{H}_i \left\{\mathbf{Z}_i^TE[\mathbf{\Sigma}_{\varepsilon_i}^{-1}](\mathbf{y}_i-E_x[b'(\sigma x + \mu)]) - \mathbf{M}_{q(\mathbf{\Sigma}_R^{-1})}\boldsymbol{\mu}_{q_{(u_i)}} - \mathbf{G}_i^T(\boldsymbol{\mu}_{q_{(\mathbf{\beta})}}-\boldsymbol{\mu}_{q_{(\mathbf{\beta})}}^{OLD}) \right\}$
13. $\mu \leftarrow \mathbf{X}\boldsymbol{\mu}_{q(\mathbf{\beta})} - \begin{bmatrix} \mathbf{Z}_{1}\boldsymbol{\mu}_{q_{(u_{1})}} \\\vdots \\ \mathbf{Z}_{m}\boldsymbol{\mu}_{q_{(u_{m})}} \end{bmatrix}$; $\quad \mathbf{\Omega} \leftarrow \sqrt{\mathbf{1}_n\mathbf{1}_8^T + \sigma(\mathbf{s}^2)^T}$; $\quad \sigma \leftarrow \text{diagonal}\{\mathbf{X}\Sigma_{q(\mathbf{\beta})}\mathbf{X}\}$
14. **for**$i = 1,\ldots, m$
15.    $\sigma_i \leftarrow \sigma_i -2\text{diagonal}\{\mathbf{Z}_{i}(\mathbf{\Sigma}_{q(\mathbf{\beta})}\mathbf{G}_{i}\mathbf{H}_{i})^T\mathbf{X}_{i}^T\} + \text{diagonal}\{\mathbf{Z}_{i}\Sigma_{q(u_{i})}\mathbf{Z}_{i}^T \}$
16. If marker $r$ is Gaussian, $E_x[b'(\sigma x + \mu)]_r \leftarrow \mu$; $\quad E_x[b''(\sigma x + \mu)]_r \leftarrow \mathbf{I}_{\sum_i n_{ir}}$
17. If marker $r$ is Poisson, $E_x[b'(\sigma x + \mu)]_r \leftarrow E_x[b''(\sigma x + \mu)]_r \leftarrow \exp \left( \mu + \frac{1}{2}\sigma \right)_r$;
18. If marker $r$ is binary, $E_x[b'(\sigma x + \mu)]_r \leftarrow \Phi\left(\frac{\mu\mathbf{s}^T}{\mathbf{\Omega}}\mathbf{p}\right)$; $\enspace E_x[b''(\sigma x + \mu)]_r \leftarrow \left\{\phi\left(\frac{\mu\mathbf{s}^T}{\mathbf{\Omega}}\right)/\mathbf{\Omega}\right\}\mathbf{p}\odot\mathbf{s}$
29. **for**$r=1,\ldots,R_c$
20.    $B_{q(\sigma^2_{\varepsilon_r})} \leftarrow \mu_{q(1/\sigma^2_{\varepsilon r})} + \frac{1}{2} \left\{ ||\mathbf{y}_r - \mathbf{C}_r\boldsymbol{\mu}_{q(\beta_r,\mathbf{u}_r)}||^2 + \text{tr}(\mathbf{C}_r^T\mathbf{C}_r\mathbf{\Sigma}_{q(\mathbf{\beta}_r,\mathbf{u}_r)})\right\} $
21.    $\mu_{q(1/\sigma^2_{\varepsilon_r})} \leftarrow \frac{\frac{1}{2}(\sum_{i=1}^m n_{ir}+1)}{B_{q(\sigma^2_{\varepsilon_r})}}$; $\, B_{q(a_{\varepsilon_r})} \leftarrow \mu_{q(1/\sigma^2_{\varepsilon_r})} + A_{\varepsilon_r}^{-2}$; $\, \mu_{q(1/a_{\varepsilon_r})} \leftarrow \frac{1}{\mu_{q(1/\sigma^2_{\varepsilon_r})} + A_{\varepsilon_r}^{-2}}$
22. **for**$k=1,\ldots,q$$B_{q(a_k)} \leftarrow \nu M_{q(\Sigma_R^{-1})_{kk}} + A_k^{-2}$; $\quad \mu_{q(1/a_k)} \leftarrow \frac{\frac{1}{2}(\nu+q)}{B_{q(a_k)}}$
23. $\mathbf{B}_{q(\mathbf{\Sigma}_R)} \leftarrow \sum^m_{i=1} \left(\boldsymbol{\mu}_{q(\mathbf{u}_i)}\boldsymbol{\mu}_{q(\mathbf{u}_i)}^T + \mathbf{\Sigma}_{q(\mathbf{u}_i)} \right) + 2\nu \text{diag}\{\mu_{q(1/a_1)}, \ldots, \mu_{q(1/a_q)}\}$
24. $\mathbf{M}_{q(\mathbf{\Sigma}_R^{-1})} \leftarrow (\nu + q + m - 1)\mathbf{B}^{-1}_{q(\mathbf{\Sigma}_R)}$
25. until the increase in $\underline{p}(\mathbf{y}, q)$ is negligible

## 4. Simulation study

In this section, we assess the performance of the MFVB Algorithm 1, through a simulation study. We are interested in two key measures of performance; the accuracy of the MFVB posterior distributions when compared to posteriors derived using MCMC, and the speed gains in using the MFVB algorithm over the MCMC algorithm.

We designed two simulation scenarios. The first considered three continuous longitudinal markers according to model (4.4). The second scenario, considered one continuous, one binary and one Poisson longitudinal marker, according to model 4.5,

(4.4) \begin{align*} Y_{i,1,j} & = 0.68 - 0.95x_{i,1,j} + u_{i11} + u_{i12}x_{i,1,j} + \varepsilon_1, & \sigma^2_{\varepsilon_1} & = 0.1,\nonumber\\ Y_{i,2,j} & = -2.50 + 0.12x_{i,2,j} + u_{i21} + u_{i22}x_{i,2,j} + \varepsilon_2, & \sigma^2_{\varepsilon_2} & = 0.25,\nonumber\\ Y_{i,3,j} & = 0.45 + 1.21x_{i,3,j} + u_{i31} + u_{i32}x_{i,3,j} + \varepsilon_3, & \sigma^2_{\varepsilon_3} & = 0.15,\nonumber\\ u & \sim N\left(\begin{bmatrix} 0 \\ 0 \\ 0 \\ 0 \\ 0 \\ 0 \end{bmatrix},\begin{bmatrix} 2.58 & 0.46 & 0.22 & 0.42 & 0.78 & 0.23 \\ 0.46 & 1.21 & 0.37 & 0.69 & 0.14 & 0.19 \\ 0.22 & 0.37 & 1.04 & 0.73 & 0.61 & 0.38 \\ 0.42 & 0.69 & 0.73 & 1.36 & 0.87 & 0.14 \\ 0.78 & 0.14 & 0.61 & 0.87 & 1.73 & 0.92 \\ 0.23 & 0.19 & 0.38 & 0.14 & 0.92 & 1.47 \end{bmatrix} \right)\!. \end{align*}

We assumed $m=(100,1000,10\,000)$ patients and simulated 100 data sets for each sample size. For each individual patient, we simulated between 5 and 10 visits according to a uniform distribution. At each visit, we simulated the three response outcome measurements according to model (4.4) or (4.5),

(4.5) \begin{align*} Y_{i,1,j} & = 0.68 - 0.95x_{i,1,j} + u_{i11} + u_{i12}x_{i,1,j} + \varepsilon_1, \nonumber\\ \log(E[Y_{i,2,j}]) & = -2.50 + 0.12x_{i,2,j} + u_{i21} + u_{i22}x_{i,2,j}, \nonumber \\ \text{logit}(E[Y_{i,3,j}]) & = 0.45 + 1.21x_{i,3,j} + u_{i31} + u_{i32}x_{i,3,j}, \end{align*}

with all other simulation details remaining unchanged. For each of the simulated data sets we first fit a MGLMM using MCMC sampling using the R package mixAK (Komárek and Komárková, 2014). We simulated 10 000 samples after a burn in of 5000 and thinned by 10. Convergence of the MCMC samples was assessed by trace plots and autocorrelation functions. We also fit a MGLMM using our streamlined MFVB algorithm. The stopping criteria for our algorithm was the relative change in the log lower bound, $\log\underline{p}(\mathbf{y}, q)$ falling below $10^{-7}$ or a maximum of 500 iterations. Each simulated data set was submitted to the University of Liverpool cluster computing system, Condor, and the computations were performed on Windows 10 computers with a 3.4 gigahertz Intel Core i7-6700 processor and 16 gigabytes of random access memory.

### 4.1. Comparison of accuracy

To compare the accuracy of the MFVB algorithm to the MCMC sample, we calculated the accuracy score based on the integrated absolute error as proposed by Faes *and others* (2011).

(4.6) \begin{equation*} \text{accuracy}(q_i^*(\theta_i)) = 100\left(1-\frac{1}{2}\int_{-\infty}^{\infty}|q_i^*(\theta_i)-p_{MCMC}(\theta|y)|\rm d\theta\right)\%. \end{equation*}

We used a kernel density estimate with plug-in bandwidth to estimate $p_{\rm MCMC}(\theta|y)$ using the R package KernSmooth, (Wand and Ripley, 2009).

**Fig. 1:**
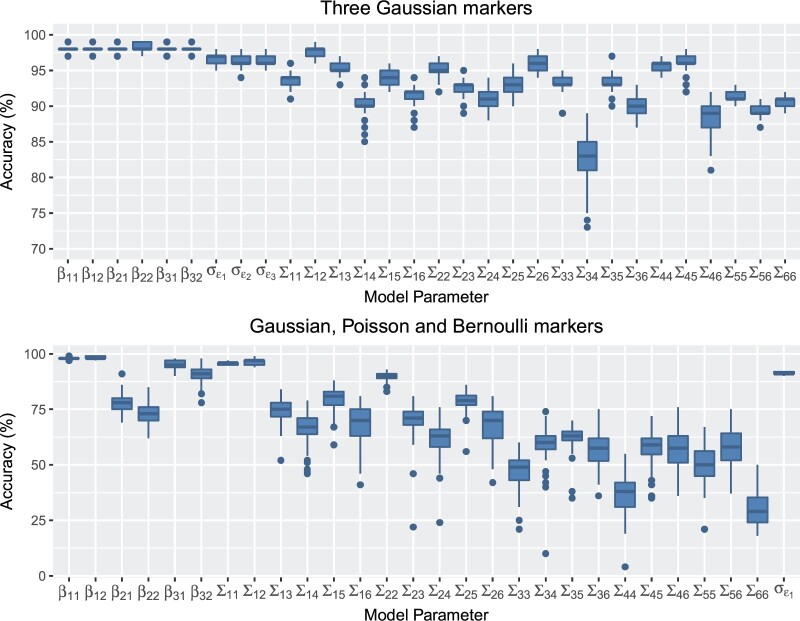
Accuracy scores for mean field variational Bayes compared to MCMC for simulated data sets with three continuous longitudinal markers (top panel) and three types of markers (bottom panel) in the simulation with $m=100$ individuals.

Figure 1 shows boxplots of the accuracy for each parameter in simulation scenarios 1 and 2, in the case where there were $m=100$ individuals. Similar plots for $m=1000$ and $m=10\,000$ individuals are shown in the Figures S1 and S2 of the Supplementary material available at *Biostatistics* online. When all the markers are continuous, the MFVB algorithm estimates the posterior distribution with very good accuracy. The fixed effects are estimated very well, as are the estimates of residual standard deviations, with very little difference between the MCMC and MFVB posteriors. The random effects covariance matrix is slightly less accurate, but the MFVB posteriors are still very similar to the MCMC posteriors. When some of the markers are non-Gaussian, the MFVB estimates are less accurate. The fixed effects are still generally well estimated although the random effects covariances less so. This is a well-known feature of MFVB algorithms (see e.g., Luts and Wand, 2015). However, an inspection of Figures S3 and S4 of the Supplementary material available at *Biostatistics* online, which show the posterior density functions for scenarios 1 and 2, respectively for a single simulated data set, shows that the means of the posterior distributions are usually very similar for both the MFVB and MCMC approaches and also that the true parameter value was usually within MFVB credible intervals. The accuracy of posterior distribution estimation does not appear to be influenced by sample size very much.

### 4.2. Comparison of computational speed

We also quantified the difference in computational time between the MFVB and MCMC approaches. The average time taken to fit each model in the simulated data sets is shown in Table 1. The MFVB routine is clearly substantially faster than the MCMC procedure. When all markers in an MGLMM are continuous, the speed gains are particularly noticeable. For example, in the model with 10 000 patients, the MCMC model takes more than 6 hours to fit, whilst the MFVB takes less than 7 min. The speed gains are less substantial in these simulations where the markers are not all continuous, although even in this case, when there are 10 000 patients, the MCMC model takes over 10.5 h to fit, whilst the MFVB model fits in just over an hour. The convergence of the MFVB algorithm is slower when adjustments need to be made for the Poisson and binary markers.

**Table 1.** Average (standard deviation) computing time in seconds, for MFVB and MCMC approaches in simulated data sets.

	MCMC	MFVB	Ratio
Three Gaussian markers			
$m=100$	267.32 (63.97)	3.79 (1.46)	70.49
$m=1000$	2029.57 (497.02)	31.28 (11.70)	64.88
$m=10000$	23 264.57 (5029.08)	400.54 (125.54)	58.08
One Gaussian, Poisson, and Bernoulli marker			
$m=100$	430.26 (167.45)	35.07 (17.35)	12.27
$m=1000$	4717.34 (1222.18)	635.45 (225.35)	7.42
$m=10\,000$	38 121.47 (15 647.86)	4009.55 (2566.41)	9.51

A comparison of speeds is to some extent subjective. Both approaches to fitting a MGLMM have different stopping criteria, which we have described at the beginning of this section. We have used freely available code to estimate our MCMC models. More efficient software could perhaps be written although, in our testing, the mixAK package was quicker than the more flexible rstan package for fitting MGLMMs using MCMC (in terms of obtaining the same number of samples with the same burn-in and thinning settings). We note too that other packages within R, such as the stan_mvmer function in rstanarmGoodrich *and others* (2018) or the brms package Bürkner (2017) could fit the models considered in this article, and bespoke codes may indeed produce MCMC estimates faster. Nevertheless, our aim in comparing speeds in this article is to show that MFVB models are substantially quicker than off-the-shelf software for MCMC.

To summarize our simulation results, we have shown that MFVB algorithms offer substantial time gains in fitting MGLMMs. These gains needs to be balanced against the reduced accuracy in some of the posterior distribution estimates, especially when not all of the markers are continuous. However, depending on which parameters are of interest to the researcher, the MFVB algorithm gives estimates of the means of the posterior distributions that are very similar to those obtained by MCMC, but in a much shorter time frame.

## 5. Real data examples

We now demonstrate the use of the MFVB algorithm to fit MGLMMs in two real data applications. The first is the well known, but relatively small primary biliary cirrhosis (PBC) data set. This data is publicly available within the mixAK package in R (and also in Appendix D of Fleming and Harrington (1991) and at http://lib.stat.cmu.edu/datasets/pbcseq). It contains measurements of seven continuous (bilirubin, albumin, alkaline phosphatase, cholesterol, serum glutamic-oxaloacetic transaminase, platelet count, and prothrombosis time) and three binary longitudinal markers (presence of ascites, hepatomegaly, and blood vessel malformations (spiders)) on 312 patients.

The second data set is a much larger data set coming from the Individualised Screening for Diabetic Retinopathy (ISDR) Cohort Study at the University of Liverpool. This study collected biomarker information on a number of risk factors for diabetic retinopathy in patients with diabetes who attended screening programs in the Merseyside region. For the purposes of this illustration, we will consider data on 17 682 patients, for whom we have repeated measurements of 10 continuous markers (HbA1c (mmol/mol), cholesterol (mmol/L), diastolic blood pressure (mm/Hg), systolic blood pressure (mm/Hg), high-density lipoprotein cholesterol (mmol/L), low-density lipoprotein cholesterol (mmol/L), eGFR (mL/min/1.73 m$^2$), Albumin-Creatinine Ratio (mg/mmol), triglycerides (mmol/L), and body mass index (kg/m$^2$) and two binary markers (retinopathy gradings in left and right eyes). A patient had a retinopathy grading of 0 if they were graded R0 (no retinopathy) in the eye being examined, and 1 if they were graded R1 (mild non-proliferative/background retinopathy). We have not considered any observations where the gradings showed more serious retinopathy, and so this analysis considers the longitudinal trajectories before sight threatening diabetic retinopathy is diagnosed. More details on this cohort can be found in García-Fiñana *and others* (2019) and Eleuteri *and others* (2017). Note that in both examples not all markers were collected at every time point for each patient. For each continuous marker, we considered a model with a random intercept and random slope, and a random intercept model for each binary marker. In addition, each marker had a fixed intercept and time slope. The values for the hyperparameters, $A_{\varepsilon_r}$, $A_k$, and $\sigma_{\beta}^2$ are each set to 10 000 and $\nu=2$. In this analysis, all continuous markers except for systolic/diastolic blood pressure, body mass index, and eGFR were log transformed. All continuous markers were then scaled prior to the analysis.

We compared the time taken to fit MGLMMs, and the accuracy of the posterior distributions for increasing numbers of markers in each data set. As before we assessed the accuracy using the integrated absolute error (4.6). All computations were performed on a personal computer with Windows 10 operating system and a 3.5 gigahertz Intel Xeon E5-1620 processor and 16 gigabyte of random access memory.

### 5.1. Primary biliary cirrhosis

We first assessed the MFVB algorithm in the PBC data which is small enough for MCMC sampling to be computationally feasible, even in the 10 marker model. Our aim was to provide *proof-of-concept* in a small data set where comparison to MCMC was relatively easy, in order to justify the use of the MFVB algorithm in much larger data sets where MCMC would be computationally challenging.

Table 2 shows the time taken to fit MGLMMs with increasing numbers of markers. In general, the MFVB approach was substantially faster than MCMC sampling. As more markers were included in the model, the improvement by using the MFVB approach was even more noticeable. Notice that even in a relatively small data set, the full 10-marker MGLMM took around 48 min to fit using MCMC, but only 14 s using our MFVB algorithm. An example of the failure to converge of the MFVB model can be seen in the 8-marker model. Nevertheless, after 500 iterations, the results, although not technically converged, still gave good accuracy estimates (results not shown, but are comparable to those presented in the 10-marker model in Figure S5 of the Supplementary material available at *Biostatistics* online.

**Table 2.** Average computing time for MFVB and MCMC approaches in primary biliary cirrhosis data (in seconds) and in the diabetic retinopathy data (in hours).

Number of markers	1	2	3	4	5	6	7	8	9	10	11	12
Primary biliary cirrhosis computation times (in seconds)												
MCMC	84.20	134.65	219.19	342.17	483.93	665.26	895.80	1983.25	2409.01	2890.92		
MFVB	1.30	2.88	3.14	5.95	6.64	6.55	6.87	27.73	13.73	14.75		
Ratio	64.77	46.80	69.74	57.53	72.92	101.58	130.39	71.52	175.43	196.05		
Diabetic retinopathy computation times (in hours)												
MCMC	1.14	1.71	2.90	4.44	6.41	8.90	11.99	15.84	19.78	24.80	53.22	62.19
MFVB	0.04	0.07	0.12	0.32	0.33	0.47	0.48	0.51	0.64	0.69	0.77	0.92
Ratio	27.56	25.22	24.08	13.66	19.59	18.97	24.89	31.16	30.77	35.68	69.02	67.57

In terms of accuracy, we present here the results for the most complicated model with 10 longitudinal markers. Figure S5 of the Supplementary material available at *Biostatistics* online shows heat maps showing the accuracy for the model parameters and the implied correlations between longitudinal markers. Only two random effects variances score lower than 50$\%$ accuracy, whilst the majority of parameters are estimated with good to excellent accuracy, showing that very similar results can be obtained in the 14 s required for the MFVB algorithm, and the MCMC sampling that required 48 min. As in the simulation studies, the fixed effects were estimated with very high accuracy.

### 5.2. Individualized screening for diabetic retinopathy

**Fig. 2:**
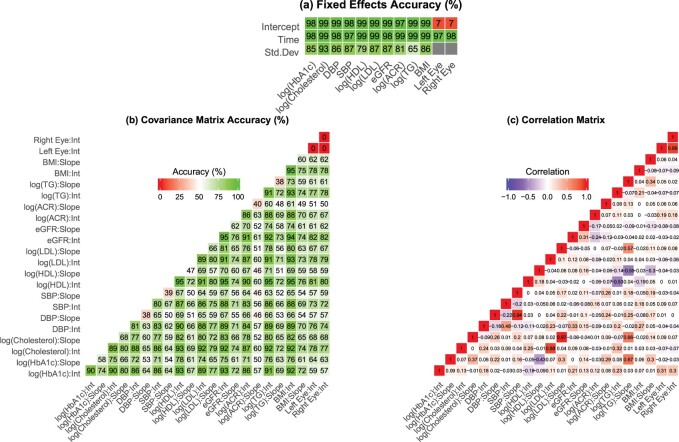
Model results for a 12 marker multivariate generalized linear mixed model in the diabetic retinopathy. Panel (a) shows the accuracy heat maps of the MFVB fixed effects estimates and residual standard deviations (compared to the MCMC estimates), (b) shows the accuracy of the MFVB random effects covariance matrix (compared to the MCMC estimates), and (c) shows the implied matrix of correlations between the 12 longitudinal markers calculated using MFVB.

The diabetic retinopathy application demonstrates the performance of MFVB in a much larger data set and gives a greater indication of the speed gains possible with MFVB. The times in hours of models with increasing numbers of longitudinal markers are shown in Table 2. The full 12-marker model was fit in less than one hour using MFVB whilst the MCMC fit required more than 2.5 days. Figure 2 reports the accuracy of this model. Again the fixed effects estimates are generally very well estimated, and most of the random effects covariance matrix parameters are reasonably accurately estimated, and clearly in a much shorter time frame than the MCMC model. The random effects for the two binary intercepts are poorly estimated in this case.

The correlation plot in panel (c) of Figure 2 reveals markers that are highly correlated, and shows why one may wish to model longitudinal markers simultaneously. We are able to identify reasonably strong positive correlations between changes over time in a patient’s triglycerides values and their HbA1c, cholesterol, and low-density lipoprotein cholesterol values. We also note negative correlations between triglycerides and high-density lipoprotein cholesterol both in terms of initial value and changes over time.

**Fig. 3:**
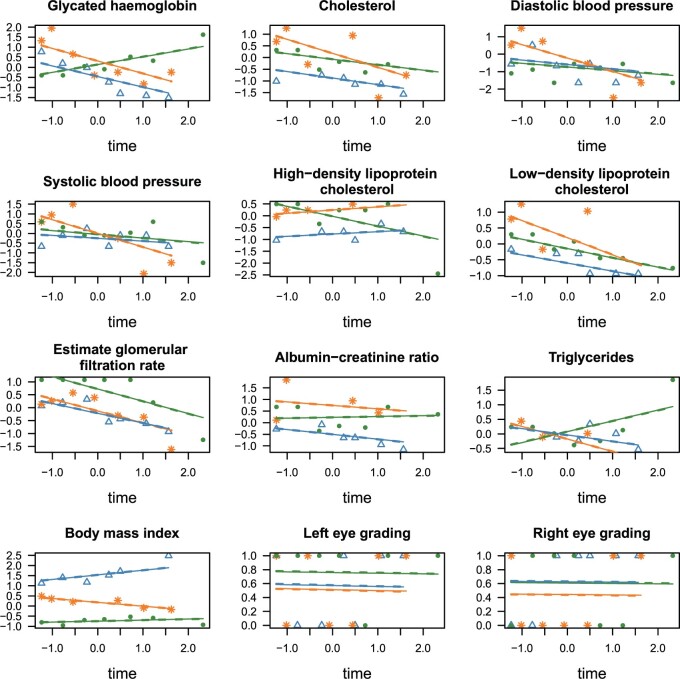
Fitted longitudinal markers for mean field variational Bayes (dashed lines) compared to MCMC (solid lines) for the 12 markers in the diabetic retinopathy data, for three patients. The orange stars, green dots, and blue triangles show the observed values for three different patients, with the respectively colored lines showing the fitted models for each individual. All continuous values, including time, have been scaled prior to analysis and the results plotted here are in terms of the scaled variables. The y-axis of each plot shows the scale version of the variable noted in the title of each panel. The original units for each variable can be found in the description at the start of Section 5.

Figure 3 shows the fitted models for each of the 12 diabetic retinopathy markers for three patients. There is very little difference between the fitted regression lines obtained by MCMC and MFVB. Even when accuracy scores (compared to MCMC) are not as high as one might desire, many of the results extracted from a model fit are almost identical to those that would be obtained with MCMC. The lower accuracy is largely caused by the known problem of poor covariance estimation for some parameters.

## 6. Summary

In this article, we present an approach for fast approximate Bayesian inference for multivariate longitudinal data. We have described how mean field variational Bayes can be used to obtain fast accurate results, that are very similar to those obtained by the much slower MCMC routines. Our article adds to the growing literature showing that MFVB is a promising avenue for fast inference in Bayesian models and demonstrates that this usefulness extends to multivariate generalized linear mixed models.

We have demonstrated through simulation studies and through application to clinical data sets that MFVB offers significant time gains over MCMC, although sometimes with the cost of less accurate estimation of covariance. This could be of use in early exploration of model fits, where assessing multiple competing models is prohibitive if models take days rather than minutes/hours to fit. MFVB could also be used to obtain good starting points for MCMC based inference, in an attempt to speed up MCMC procedures. However, we believe our article demonstrates that for many outputs of interest, MFVB provides good estimates in its own right.

Future work should investigate ways to improve the speed of MFVB algorithms further, without losing accuracy in the estimation of posterior distributions. One possible avenue for pursuing this could be through model reparameterization which Tan (2021) shows can improve both accuracy and speed of convergence.

We have demonstrated our MFVB approach in two clinical data sets. Although the diabetic retinopathy data consists of data on 12 longitudinal markers for 17 682 patients, this number is potentially small in comparison to the data increasingly available form sources such as electronic health records, where data may be held on hundreds of thousands of patients, with many more than 12 longitudinal markers. Although MCMC is slow in the diabetic retinopathy application (with the 12-marker model taking more than 2.5 days to fit), it is still at least feasible. This would not be the case in the much larger data sets available through electronic health records.

In this article, we have shown that MFVB can give accurate parameter estimates in much faster times, which gives confidence that they could do so in settings where MCMC was computationally infeasible. In this case, it is desirable to have some indication about how good an MFVB approximation is. Two promising *post hoc* diagnostic tools have been proposed to assess goodness of fit by Yao *and others* (2018). The first assesses the goodness of fit of the joint distribution (i.e., how close is $q(\mathbf{\theta})$ to the true $p(\mathbf{\theta}|y)$), interpreting the shape parameter from Pareto smoothed importance sampling as the Renyi divergence between $q(\mathbf{\theta})$ and $p(\mathbf{\theta}|y)$, with small divergences indicating good fit. This approach offers the interesting prospect of *correcting* MFVB estimates post analysis and would be a profitable avenue for further research. The second diagnostic proposed is a variational simulation based calibration diagnostic that assesses the average performance of point estimates from an MFVB approximation.

The applications to primary biliary cirrhosis and diabetic retinopathy data in this article were for the purposes of illustration. One may also wish to consider the influence of many other covariates on the longitudinal profiles of various markers. This is perfectly possible within the algorithm presented in this article. Similarly, as not all markers are measured at each time point, there could well be information simply in the fact that a marker was measured. Additional work could be done to model this informative observation. This was outside the scope of this article and would be an interesting avenue for future work.

The problem of poorly estimated covariance matrices observed in this article is a well-known problem with MFVB algorithms. How much of a problem this is depends on what a researcher wants from a model. If estimates of posterior means are required then MFVB can provide very good estimates. Equally, if the MGLMM is to be used for prediction or classification (e.g., Hughes *and others*, 2018) then fast and accurate estimate of posterior means may be sufficient. If a more accurate assessment of variability is required, then more work is required. One promising area we are currently investigating is the use of linear response variational Bayes to *correct* MFVB variance estimates (Giordano *and others*, 2015).

Although we have shown in this article that MFVB can provide a very useful modelling tool in complex longitudinal models, there is no guarantee that MFVB will always provide a good solution. Much depends on how much correlation is ignored in the mean field product restriction. Additionally, Nolan and Wand (2017) show that the amount of posterior correlation between regression parameters can affect the performance of MFVB. Other features of a problem, unrelated to MFVB specifically, such as the number of repeated measurements per individual, and the sample size in general will likely contribute to the quality of a MFVB approximation.

Overall, MFVB offers a fast and useful alternative to MCMC for scalable Bayesian inference in complex longitudinal data.

## 7. Software

Software in the form of R code, together with a sample input data set and complete documentation is available on request from the corresponding author (dmhughes@liverpool.ac.uk). Code to reproduce the PBC analysis is available on GitHub at https://github.com/dmhughesLiv/VariationalBayes

## Supplementary Material

kxab021_Supplementary_Data
