# Supplementary material for: Fast approximate inference for multivariate longitudinal data
Source: Biostatistics. 2021 May 15;24(1):177–92. doi: 10.1093/biostatistics/kxab021 (PMC9748580; doi:10.1093/biostatistics/kxab021)
Supplement: kxab021_Supplementary_Data [file kxab021_supplementary_data.pdf]

Biostatistics (2021), **0**, 0, *pp.* **??**–16
doi:10.1093/biostatistics/Hughesetal˙MFVBmglmm˙Biostatistics˙Supplement

Supplement to: Fast approximate inference for multivariate longitudinal data

DAVID M. HUGHES*, MARTA GARCÍA-FIÑANA

Department of Health Data Science, University of Liverpool, United Kingdom

dmhughes@liverpool.ac.uk

MATT P. WAND

School of Mathematical and Physical Sciences, University of Technology Sydney, Australia

Summary

This document gives details of the derivation of the mean field variational Bayes (MFVB) approximation to a multivariate generalised linear model. The optimal form of each q-density is derived, and an expression for the log lower bound on the marginal log-likelihood is given. Additional simulation results are also provided.

Key words: Multivariate mixed models; Mean field variational Bayes; Generalised linear mixed model; Bayesian computing; Markov chain Monte Carlo; Repeated measurements.

*To whom correspondence should be addressed.

© The Author 2021. Published by Oxford University Press. All rights reserved. For permissions, please e-mail: journals.permissions@oup.com

1. Derivation of optimal q^* densitites

To find optimal densities for each part of the factorisation of $q(\theta)$ we use the following result.

$$q_i^*(\theta_i) \propto \exp\{E_{-\theta_i}[\log p(\mathbf{y}, \theta)]\} \tag{1.1}$$

For the MGLMM presented in the main manuscript, we have

$$\begin{aligned} E_{-\theta_i}[\log p(\mathbf{y}, \theta)] = E_{-\theta_i}[\log p(\mathbf{y}, |\beta, \mathbf{u}, \mathbf{\Sigma}_\varepsilon)] + E_{-\theta_i}[\log p(\beta, \mathbf{u}|\mathbf{\Sigma}_R)] + E_{-\theta_i}[\log p(\mathbf{\Sigma}_R|a_1, \ldots, a_q)] \\ + \sum_{k=1}^{q} E_{-\theta_i}[\log p(a_k)] + \sum_{r=1}^{R_c} \left\{ E_{-\theta_i}\left[\log p(\sigma^2_{\varepsilon_r}|a_{\varepsilon_r})\right] + E_{-\theta_i}[\log p(a_{\varepsilon_r})] \right\} \end{aligned}$$

1.1 *Derivation of* $q^*(\sigma^2_{\epsilon r})$

We require for each $r = 1, \ldots, R_c$

$$\begin{aligned} E_{-\sigma^2_{\epsilon_r}}[\log p(\mathbf{y}, \theta)] &= E_{-\sigma^2_{\epsilon_r}}[\log p(\mathbf{y}, |\beta, \mathbf{u}, \mathbf{\Sigma}_\varepsilon)] + E_{-\sigma^2_{\varepsilon_r}}[\log p(\sigma^2_{\varepsilon_r}|a_{\varepsilon_r})] + \text{const} \\ &= -\frac{1}{2} E_{-\sigma^2_{\varepsilon_r}}[\log|\mathbf{\Sigma}_\varepsilon|] - \frac{1}{2} E_{-\sigma^2_{\varepsilon_r}}[(\mathbf{y} - \mathbf{C}\tilde{\nu})^T \mathbf{\Sigma}_\varepsilon^{-1} (\mathbf{y} - \mathbf{C}\tilde{\nu})] - \frac{3}{2}\log \sigma^2_{\varepsilon_r} \\ &- \frac{1}{\sigma^2_{\varepsilon_r}} E_{-\sigma^2_{\varepsilon_r}}[1/a_{\varepsilon_r}] + \text{const} \\ &= -\left(\frac{\frac{1}{2}(\|\mathbf{y}_r - \mathbf{C}_r \mu_{q(\beta_r, \mathbf{u}_r)}\|^2 + \text{tr}[\mathbf{C}_r^T \mathbf{C}_r \mathbf{\Sigma}_{q(\beta_r, \mathbf{u}_r)}]) + \mu_{q(1/a_{\varepsilon_r})}}{\sigma^2_{\varepsilon_r}} \right) \\ &\left\{ -\frac{1}{2}\left(\sum_{i=1}^{m} n_{ir} + 1 \right) - 1 \right\} \log \sigma^2_{\varepsilon_r} + \text{const} \end{aligned}$$

Here, the subscript r denotes the rows of the overall model relevant to the rth continuous longitudinal marker. Hence, $q^*(\sigma^2_{\varepsilon r})$ is an inverse-Gamma$(1/2(\sum_{i=1}^{m} n_{ir} + 1), B_{q(\sigma^2_{\varepsilon_r})})$ distribution with

$$B_{q(\sigma^2_{\varepsilon_r})} = \mu_{q(1/\sigma^2_{\varepsilon r})} + \frac{1}{2}\left\{ ||y_r - C_r \mu_{q(\beta_r, u_r)}||^2 + tr(C_r^T C_r \Sigma_{q(\beta_r, u_r)}) \right\}$$

1.2 *Derivation of* $q^*(a_{\varepsilon r})$

We require for each $r = 1, \ldots, R_c$,

$$\begin{aligned}
E_{-a_{\varepsilon_r}}[\log p(\mathbf{y}, \theta)] &= E_{-a_{\varepsilon_r}}[\log p(\sigma^2_{\varepsilon_r}|a_{\varepsilon_r})] + E_{-a_{\varepsilon_r}}[\log p(a_{\varepsilon_r})] + \text{const} \\
&= -2\log a_{\varepsilon_r} - \frac{\left(E_{-a_{\varepsilon_r}}[\frac{1}{\sigma^2_{\varepsilon_r}}] + A^{-2}_{\varepsilon_r}\right)}{a_{\varepsilon_r}} + \text{const}
\end{aligned}$$

Hence $q^*(a_{\varepsilon r})$ is an inverse-Gamma$(1, B_{q(a_{\varepsilon r})})$ distribution with

$$B_{q(a_{\varepsilon r})} = \mu_{q(1/\sigma^2_{\varepsilon r})} + A^{-2}_{\varepsilon r}.$$

1.3 *Derivation of* $q^*(a_k)$

We require for each $k = 1, \ldots, q$,

$$\begin{aligned}
E_{-a_k}[\log p(\mathbf{y}, \theta)] &= E_{-a_k}[\log p(\mathbf{\Sigma}_R|a_1, \ldots, a_q)] + E_{-a_k}[\log p(a_k)] + \text{const} \\
&= -\frac{(\nu + q + 2}{2}\log(a_k) - \frac{E_{-a_k}[\mathbf{\Sigma}_R]_{kk} + A_k^{-2}}{a_k} + \text{const} \\
&= -\frac{(\nu + q + 2}{2}\log(a_k) - \frac{\nu \mathbf{M}_{q(\Sigma_R^{-1})_{kk}} + A_k^{-2}}{a_k} + \text{const}
\end{aligned}$$

So $q^*(a_k)$ is an inverse-Gamma$(1/2(\nu + q), B_{q(a_k)})$ with

$$B_{q(a_k)} = \nu \mathbf{M}_{q(\Sigma_R^{-1})_{kk}} + A_k^{-2}.$$

1.4 *Derivation of* $q^*(\mathbf{\Sigma}_R)$

We require

$$
\begin{aligned}
E_{-\mathbf{\Sigma}_R}[\log p(\mathbf{y},\theta)] &= E_{-\mathbf{\Sigma}_R}[\log p(\mathbf{u}|\mathbf{\Sigma}_R)] + E_{-\mathbf{\Sigma}_R}[\log p(\mathbf{\Sigma}_R|a_1,\ldots,a_q)] + \text{const}\\
&= -\frac{(m+\nu+2q)}{2}\log|\mathbf{\Sigma}_R| - \frac{1}{2}E_{-\mathbf{\Sigma}_R}[\mathbf{u}^T(I_m\otimes\mathbf{\Sigma}_R^{-1})\mathbf{u}]\\
&- E_{-\mathbf{\Sigma}_R}[\frac{1}{2}\text{tr}(2\nu\text{diag}\{1/a_1,\ldots,1/a_q\}\mathbf{\Sigma}_R^{-1})] + \text{const}\\
&= -\frac{1}{2}\text{tr}\left(\left\{\sum_{i=1}^{m}(\mu_{q(u_i)}\mu_{q(u_i)}^T + \Sigma_{q(u_i)}) + 2\nu\text{diag}(\mu_{q(1/a_1)},\ldots,\mu_{q(1/a_q)})\right\}\mathbf{\Sigma}_R^{-1}\right)\\
&- \frac{(m+\nu+2q)}{2}\log|\mathbf{\Sigma}_R| + \text{const}
\end{aligned}
$$

Hence $q^*(\mathbf{\Sigma}_R)$ is an inverse-Wishart$(\nu+q+m-1, B_{q(\Sigma_R)})$ with

$$
B_{q(\Sigma_R)} = \sum_{i=1}^{m}(\mu_{q(u_i)}\mu_{q(u_i)}^T + \Sigma_{q(u_i)}) + 2\nu\text{diag}(\mu_{q(1/a_1)},\ldots,\mu_{q(1/a_q)})
$$

1.5 *Derivation of* $q^*(\beta,\mathbf{u})$

When all of the longitudinal markers are continuous (and assumed Gaussian), it is relatively trivial to show the $q^*(\beta,\mathbf{u})$ is multivariate normal. However, once any of the longitudinal markers are binary or counts (assumed to be Poisson), we can no longer obtain a nice distribution for $q^*(\beta,\mathbf{u})$. Instead we take the approach of Rohde and Wand (2016) and specify that

$$
q^*(\beta,\mathbf{u},\mu_{q(\beta,\mathbf{u})},\mathbf{\Sigma}_{q(\beta,\mathbf{u})} \sim N(\mu_{q(\beta,\mathbf{u})},\mathbf{\Sigma}_{q(\beta,\mathbf{u})})
$$

in order to gain tractibility. By result 2 of Rohde and Wand (2016), the necessary updates for $\mu_{q_{(\beta,\mathbf{u})}}$ and $\mathbf{\Sigma}_{q(\beta,\mathbf{u})}$ are

$$
\begin{aligned}
\nu_{q(\beta,\mathbf{u})} &\leftarrow D_{\mu_{q(\beta,\mathbf{u})}}\text{Non Entropy}(q,\mu_{q(\beta,\mathbf{u})},\mathbf{\Sigma}_{q_{(\beta,\mathbf{u})}})^T\\
\mathbf{\Sigma}_{q(\beta,\mathbf{u})} &\leftarrow -\left\{H_{\mu_{q(\beta,\mathbf{u})}}\text{Non Entropy}(q,\mu_{q(\beta,\mathbf{u})},\mathbf{\Sigma}_{q(\beta,\mathbf{u})})\right\}^{-1}\\
\mu_{q(\beta,\mathbf{u})} &\leftarrow \mu_{q(\beta,\mathbf{u})} + \mathbf{\Sigma}_{q(\beta,\mathbf{u})}\nu_{q(\beta,\mathbf{u})}
\end{aligned}
$$

where $D_x f$ is the derivate vector of f with respect to x and $H_x f$ is the Hessian matrix of f, the second derivative with respect to x, and

$$
\begin{aligned}
\text{Non Entropy}(q, \mu_{q(\beta,\mathbf{u})}, \boldsymbol{\Sigma}_{q(\beta,\mathbf{u})}) &= E_q\left[\log p(\mathbf{y}, |\beta, \mathbf{u}, \boldsymbol{\Sigma}_\varepsilon)\right] + E_q\left[\log p(\beta, \mathbf{u}|\boldsymbol{\Sigma}_R)\right] \\
&= \mathbf{y}^T E[\boldsymbol{\Sigma}_\varepsilon^{-1}]\mathbf{C}\mu_{q(\beta,\mathbf{u})} - \mathbf{1}^T E[\boldsymbol{\Sigma}_\varepsilon^{-1}]E_q[b(\mathbf{C}\tilde{\nu})] + \mathbf{1}^T E_q[c(\mathbf{y}, \phi)] \\
&- \frac{(p+mq)}{2}\log 2\pi - \frac{1}{2}E_q\left[\log\left|\begin{matrix}\sigma_\beta^{-2} I_p & 0 \\ 0 & I_m \otimes E[\boldsymbol{\Sigma}_R^{-1}]\end{matrix}\right|\right] \\
&- \frac{1}{2}\left\{\mu_{q(\beta,\mathbf{u})}^T \begin{bmatrix}\sigma_\beta^{-2} I_p & 0 \\ 0 & I_m \otimes E[\boldsymbol{\Sigma}_R^{-1}]\end{bmatrix}\mu_{q(\beta,\mathbf{u})}\right. \\
&\left. + \text{tr}\left(\begin{bmatrix}\sigma_\beta^{-2} I_p & 0 \\ 0 & I_m \otimes E[\boldsymbol{\Sigma}_R^{-1}]\end{bmatrix}\boldsymbol{\Sigma}_{q(\beta,\mathbf{u})}\right)\right\} \qquad (1.2)
\end{aligned}
$$

in our case. See Rohde and Wand (2016) for a more general definition of non-entropy. In the following, we will make use of a change of variable. Note that by our specification, $\mathbf{C}\tilde{\nu} \sim N(\mathbf{C}\mu_{q(\beta,\mathbf{u})}, \mathbf{C}\boldsymbol{\Sigma}_{q(\beta,\mathbf{u})}\mathbf{C}^T) = N(\mu, \sigma^2)$. Then we define a new variable, $x = (\mathbf{C}\tilde{\nu} - \mu)/\sigma$, and we have that $x \sim N(0, 1)$. Then, from 1.2, and using rules 3.3.6 and 3.3.2 of Wand (2002) we have that,

$$
\begin{aligned}
D_{\mu_{q(\beta,\mathbf{u})}}\text{Non Entropy}(q, \mu_{q(\beta,\mathbf{u})}, \boldsymbol{\Sigma}_{q(\beta,\mathbf{u})}) &= (\mathbf{y} - D_{\mu_{q(\beta,\mathbf{u})}}\{E_q[b(\mathbf{C}\tilde{\nu})]\})^T E[\boldsymbol{\Sigma}_\varepsilon^{-1}]\mathbf{C} \\
&- \mu_{q(\beta,\mathbf{u})}^T \begin{bmatrix}\sigma_\beta^{-2} I_p & 0 \\ 0 & I_m \otimes E[\boldsymbol{\Sigma}_R^{-1}]\end{bmatrix} \\
&= (\mathbf{y} - E_x[b'(\sigma x + \mu)])^T E[\boldsymbol{\Sigma}_\varepsilon^{-1}]\mathbf{C} - \mu_{q(\beta,\mathbf{u})}^T \begin{bmatrix}\sigma_\beta^{-2} I_p & 0 \\ 0 & I_m \otimes E[\boldsymbol{\Sigma}_R^{-1}]\end{bmatrix}
\end{aligned}
$$

and

$$
\begin{aligned}
H_{\mu_{q(\beta,\mathbf{u})}}\text{Non Entropy}(q, \mu_{q(\beta,\mathbf{u})}, \boldsymbol{\Sigma}_{q(\beta,\mathbf{u})}) &= D_{\mu_{q(\beta,\mathbf{u})}}\left\{D_{\mu_{q(\beta,\mathbf{u})}}\text{Non Entropy}(q, \mu_{q(\beta,\mathbf{u})}, \boldsymbol{\Sigma}_{q(\beta,\mathbf{u})})^T\right\} \\
&= D_{\mu_{q(\beta,\mathbf{u})}}\left\{\mathbf{C}^T E[\boldsymbol{\Sigma}_\varepsilon^{-1}](\mathbf{y} - E_x[b'(\sigma x + \mu)])\right. \\
&\left. - \begin{bmatrix}\sigma_\beta^{-2} I_p & 0 \\ 0 & I_m \otimes E[\boldsymbol{\Sigma}_R^{-1}]\end{bmatrix}\mu_{q(\beta,\mathbf{u})}\right\} \\
&= -\mathbf{C}^T E[\boldsymbol{\Sigma}_\varepsilon^{-1}]D_{\mu_{q(\beta,\mathbf{u})}}\{E_x[b'(\sigma x + \mu)]\} - \begin{bmatrix}\sigma_\beta^{-2} I_p & 0 \\ 0 & I_m \otimes E[\boldsymbol{\Sigma}_R^{-1}]\end{bmatrix} \\
&= -\mathbf{C}^T E[\boldsymbol{\Sigma}_\varepsilon^{-1}]E_x[\text{diag}(b''(\sigma x + \mu))]\mathbf{C} - \begin{bmatrix}\sigma_\beta^{-2} I_p & 0 \\ 0 & I_m \otimes E[\boldsymbol{\Sigma}_R^{-1}]\end{bmatrix}
\end{aligned}
$$

Hence the Rohde and Wand updates for $q^*(\beta, \mathbf{u})$ in the MGLMM defined in our paper are given by

$$\begin{aligned}
\nu_{q(\beta,\mathbf{u})} &\leftarrow \mathbf{C}^T E[\mathbf{\Sigma}_\varepsilon^{-1}](y - E_x[b'(\sigma x+\mu)]) - \begin{bmatrix} \sigma_\beta^{-2} I_p & 0 \\ 0 & I_m \otimes E[\mathbf{\Sigma}_R^{-1}] \end{bmatrix} \mu_{q(\beta,\mathbf{u})} \\
\mathbf{\Sigma}_{q(\beta,\mathbf{u})} &\leftarrow \left\{ \mathbf{C}^T E[\mathbf{\Sigma}_\varepsilon^{-1}] \mathrm{diag} E_x[b''(\sigma x+\mu)] \mathbf{C} + \begin{bmatrix} \sigma_\beta^{-2} I_p & 0 \\ 0 & I_m \otimes E[\mathbf{\Sigma}_R^{-1}] \end{bmatrix} \right\}^{-1} \\
\mu_{q(\beta,\mathbf{u})} &\leftarrow \mu_{q(\beta,\mathbf{u})} + \mathbf{\Sigma}_{q(\beta,\mathbf{u})} \nu_{q(\beta,\mathbf{u})}
\end{aligned}$$

It remains to define the cumulant functions and derivatives $b(\cdot)$, $b'(\cdot)$ and $b''(\cdot)$ for each of Gaussian, Poisson, and binary longitudinal markers. Note that the derivative $b'(\sigma x+\mu)$ is taken with respect to $(\sigma x+\mu)$. The chain rule is applied in the overall notation.

If $\mathbf{Y}_r$ is a Gaussian marker, then $b(\sigma x+\mu) = 1/2(\sigma x+\mu)^2$, and so $E_x[b'(\sigma x+\mu)] = \sigma E_x[x]+\mu = \mu = \mathbf{C}\mu_{q(\beta,\mathbf{u})}$, and $E_x[b''(\sigma x+\mu)] = \mathbf{1}$.

If $\mathbf{Y}_r$ is a Poisson marker, then $b(\sigma x+\mu) = b'(\sigma x+\mu) = b''(\sigma x+\mu) = e^{\sigma x+\mu}$ and so $E_x[b'(\sigma x+\mu)] = E_x[b''(\sigma x+\mu)] = \exp(\mathbf{C}\mu_{q(\beta,\mathbf{u})} + 1/2\mathrm{diagonal}(\mathbf{C}\mathbf{\Sigma}_{q(\beta,\mathbf{u})}\mathbf{C}^T))$.

If $\mathbf{Y}_r$ is a binary marker, then $b(\sigma x+\mu) = \log(1+e^{\sigma x+\mu})$. Defining $\mathrm{expit}(x) = \mathrm{logit}^{-1}(x) = 1/(1+\exp(-x))$, we have that $b'(\sigma x+\mu) = \mathrm{expit}(\sigma x+\mu)$ and $b''(\sigma x+\mu) = b'(\sigma x+\mu)/(1+\exp(\sigma x+\mu))$. It then follows that

$$E_x[b'(\sigma x+\mu)] = \int_{-\infty}^{\infty} \mathrm{expit}(\sigma x+\mu)\phi(x)dx = \mathbf{B}_0(\mu, \sigma^2)$$

and with the help of integration by parts,

$$\begin{aligned}
E_x[b''(\sigma x+\mu)] &= \int_{-\infty}^{\infty} \frac{b'(\sigma x+\mu)}{(1+\exp(\sigma x+\mu))} \\
&= \frac{1}{\sigma}\int_{-\infty}^{\infty} x \mathrm{expit}(\sigma x+\mu)\phi(x)dx \\
&= \mathbf{B}_1(\mu, \sigma^2)
\end{aligned}$$

Following Nolan and Wand (2017) we avoid quadrature routines by approximating $\mathbf{B}_0(\mu, \sigma^2)$ and $\mathbf{B}_1(\mu, \sigma^2)$ using a weighted mixture of normal distribution using the Monahan-Stefanski weights

with $k = 8$ mixture components. Nolan and Wand (2017) state that

$$\mathbf{B}_0(\mu,\sigma^2) \approx \sum_{i=1}^{k} p_{k,i}\Phi\left(\frac{\mu s_{k,i}}{\sqrt{1+\sigma^2 s_{k,i}^2}}\right)$$
$$\mathbf{B}_1(\mu,\sigma^2) \approx \sigma \sum_{i=1}^{k} \frac{p_{k,i}s_{k,i}}{\sqrt{1+\sigma^2 s_{k,i}^2}}\phi\left(\frac{\mu s_{k,i}}{\sqrt{1+\sigma^2 s_{k,i}^2}}\right)$$

In our case then the necessary MFVB updates are given by

$$\mathbf{\Omega} \leftarrow \sqrt{\mathbf{1}_n\mathbf{1}_8^T + \mathbf{C}\mathbf{\Sigma}_{q(\beta,\mathbf{u})}\mathbf{C}^T(\mathbf{s}^2)^T}$$
$$\tilde{\mathbf{B}}_0 \leftarrow \Phi\left(\frac{\mathbf{C}\mu_{q(\beta,\mathbf{u})}\mathbf{s}^T}{\mathbf{\Omega}}\mathbf{p}\right)$$
$$\tilde{\mathbf{B}}_1 \leftarrow \left\{\phi\left(\frac{\mathbf{C}\mu_{q(\beta,\mathbf{u})}\mathbf{s}^T}{\mathbf{\Omega}}\right)/\mathbf{\Omega}\right\}\mathbf{p}\odot\mathbf{s}$$

The updates for each marker can be substituted back into the Rohde and Wand updates using the relevant rows for each marker.

2. Streamlining

The updates for $\mathbf{\Sigma}_{q(\beta,\mathbf{u})}$ involve calculating the inverse of a large matrix. However, this matrix has a block sparse structure, and we can utilise this structure, and matrix algebra results, in order to streamline our algorithm, and make substantial speed gains. This streamlining procedure is outlined in more detail in Lee and Wand (2016), and we just give the relevant details for our

model here. The inverse of $\boldsymbol{\Sigma}_{q(\beta,\mathbf{u})}$ is arranged as follows.

$$\boldsymbol{\Sigma}_{q(\beta,\mathbf{u})}^{-1} = \begin{bmatrix} F & G_1 & \dots & \dots & G_m \\ G_1^T & H_1^{-1} & 0 & \dots & 0 \\ \vdots & 0 & \ddots & \vdots & \vdots \\ \vdots & \vdots & \vdots & \ddots & \vdots \\ G_m^T & 0 & \dots & 0 & H_m^{-1} \end{bmatrix}$$

$$\begin{aligned}
\text{with } F &= X^T E[\boldsymbol{\Sigma}_{\varepsilon}^{-1}]\text{diag}\{E_x[b''(\sigma x + \mu)]\}X + \sigma_{\beta}^{-2} I_p \\
G_i &= X_i^T E[\boldsymbol{\Sigma}_{\varepsilon_i}^{-1}]\text{diag}\{E_x[b''(\sigma x + \mu)]\}_i Z_i \\
H_i &= \left\{ Z_i^T E[\boldsymbol{\Sigma}_{\varepsilon_i}^{-1}]\text{diag}\{E_x[b''(\sigma x + \mu)]\}_i Z_i + M_{q(\Sigma_R^{-1})} \right\} \\
\mu &= \mathbf{C}\mu_{q(\beta,\mathbf{u})} \\
\sigma^2 &= \mathbf{C}\boldsymbol{\Sigma}_{q(\beta,\mathbf{u})}\mathbf{C}^T
\end{aligned}$$

Using corollary 8.5.12 of Harville (2008), the inverse can be found as

$$\begin{aligned}
\boldsymbol{\Sigma}_{q(\beta,\mathbf{u})} &= \begin{bmatrix} \Sigma_{q(\beta)} & \Lambda_{q(\beta,\mathbf{u})} \\ \Lambda_{q(\beta,\mathbf{u})}^T & \Sigma_{q(u)} \end{bmatrix} \\
\text{where } \Sigma_{q(\beta)} &= \left(F - \sum_{i=1}^{m} G_i H_i G_i^T \right)^{-1} \\
\Lambda_{q(\beta,\mathbf{u})} &= -\left[\Sigma_{q(\beta)} G_1 H_1, \dots, \Sigma_{q(\beta)} G_m H_m \right] \\
\Sigma_{q(u_i)} &= H_i + H_i G_i^T \Sigma_{q(\beta)} G_i H_i
\end{aligned}$$

The updates involving $\boldsymbol{\Sigma}_{q(\beta,\mathbf{u})}$ can now be streamlined which largely involves many calculations being performed only on blocks of the data containing the rows for a particular individual and so substantially improves computation time. Specifically, simple algebraic manipulations result in the updates for $\mu_{q(\beta,\mathbf{u})}$ and $B_{q(\sigma^2_{\epsilon_r})}$ given in Algorithm 1 of our main manuscript. We can also streamline the calculation of $1/2 \log |\boldsymbol{\Sigma}_{q(\beta,\mathbf{u})}|$ which occurs in the marginal log lower bound $\log \underline{p}(\mathbf{y}, q)$, using Theorem 13.3.8 of Harville (2008).

3. Derivation of a lower bound on the marginal log-likelihood

We control the convergence of our MFVB algorithm through monitoring the bound on the marginal log-likelihood, $\log \underline{p}(\mathbf{y}, q)$. This is calculated as follows.

$$\begin{aligned}
\log \underline{p}(\mathbf{y}, q) &= E_q[\log p(\mathbf{y}, \theta) - \log q(\theta)] \\
&= E_q[\log p(\mathbf{y}_G|\beta, \mathbf{u}, \mathbf{\Sigma}_\varepsilon)] + E_q[\log p(\mathbf{y}_P|\beta, \mathbf{u})] + E_q[\log p(\mathbf{y}_B|\beta, \mathbf{u})] + E_q[\log p(\beta, \mathbf{u}|\mathbf{\Sigma}_R)] \\
&+ E_q[\log p(\mathbf{\Sigma}_R|a_1, \ldots, a_q)] + \sum_{r=1}^{R_c} \left\{ E_q[\log p(\sigma^2_{\varepsilon_r}|a_{\varepsilon_r})] + E_q[\log p(a_{\varepsilon_r})] \right\} + \sum_{k=1}^{q} E_q[\log p(a_k)] \\
&- E_q[\log q(\beta, \mathbf{u})] - E_q[\log q(\mathbf{\Sigma}_R)] - \sum_{r=1}^{R_c} \left\{ E_q[\log q(\sigma^2_{\epsilon r})] + E_q[\log q(a_{\varepsilon r})] \right\} - \sum_{k=1}^{q} E_q[\log q(a_k)]
\end{aligned}$$

Here, we use $\mathbf{y}_G$, $\mathbf{y}_P$ and $\mathbf{y}_B$ to denote all the stacked Gaussian, Poisson and binary longitudinal observations respectively. The number of observations of each type of marker are denoted by n_G, n_P and n_B respectively. We also make use of matrices $\mathbf{E}_1$, $\mathbf{E}_2$ and $\mathbf{E}_3$ as $n \times n$ diagonal matrices with value 1 for rows corresponding to Gaussian, Poisson and binary markers respectively and 0 elsewhere. We now provide an expression for each part of $\log \underline{p}(\mathbf{y}, q)$ and then state the full

expression.

$$
\begin{aligned}
E_q[\log p(\mathbf{y}_G|\beta,\mathbf{u},\boldsymbol{\Sigma}_\varepsilon)] &= -\frac{n_G}{2}\log(2\pi) - \frac{1}{2}E_q[\log|\boldsymbol{\Sigma}_\varepsilon|] - \frac{1}{2}E_q[(\mathbf{y}-\mathbf{C}\tilde{\nu})^T\mathbf{E}_1^T\boldsymbol{\Sigma}_\varepsilon^{-1}\mathbf{E}_1(\mathbf{y}-\mathbf{C}\tilde{\nu})] \\
E_q[\log p(\mathbf{y}_P|\beta,\mathbf{u})] &= \mathbf{y}^T\mathbf{E}_2\mathbf{C}\mu_{q(\beta,\mathbf{u})} - \mathbf{1}^T\mathbf{E}_2\log\mathbf{y}! - \mathbf{1}^T\mathbf{E}_2\exp(\mathbf{C}\mu_{q(\beta,\mathbf{u})} + 1/2\text{diagonal}(\mathbf{C}\boldsymbol{\Sigma}_{q(\beta,\mathbf{u})}\mathbf{C}^T)) \\
E_q[\log p(\mathbf{y}_B|\beta,\mathbf{u})] &= \mathbf{y}^T\mathbf{E}_3\mathbf{C}\mu_{q(\beta,\mathbf{u})} - E_q[\mathbf{1}^T\mathbf{E}_3\log(1+\exp(\mathbf{C}\tilde{\nu}))] \\
E_q[\log p(\beta,\mathbf{u}|\boldsymbol{\Sigma}_R)] &= \frac{-(p+mq)}{2}\log(2\pi) - \frac{1}{2}E_q\left[\log\left|\begin{matrix}\sigma_\beta^{-2}\mathbf{I}_p & 0 \\ 0 & \mathbf{I}_m\otimes E[\boldsymbol{\Sigma}_R^{-1}]\end{matrix}\right|\right] \\
&\quad - \frac{1}{2}E_q\left[\tilde{\nu}^T\begin{bmatrix}\sigma_\beta^{-2}I_p & 0 \\ 0 & I_m\otimes E[\boldsymbol{\Sigma}_R^{-1}]\end{bmatrix}\tilde{\nu}\right] \\
E_q[\log p(\boldsymbol{\Sigma}_R|a_1,\ldots,a_q)] &= -\log C_{q,\nu+q-1} + \frac{\nu+q-1}{2}E_q[\log|2\nu\text{diag}\{1/a_1,\ldots,1/a_q\}|] \\
&\quad - \frac{\nu+2q}{2}E_q[\log|\boldsymbol{\Sigma}_R|] - \nu E_q[\text{tr}(\text{diag}\{1/a_1,\ldots,1/a_q\}\boldsymbol{\Sigma}_R^{-1})] \\
E_q[\log p(\sigma^2_{\varepsilon_r}|a_{\varepsilon_r})] &= -\frac{1}{2}E_q[\log(a_{\varepsilon_r})] - \log\Gamma(1/2) - \frac{3}{2}E_q[\log\sigma^2_{\varepsilon_r}] - E_q[1/a_{\varepsilon_r}\sigma^2_{\varepsilon_r}] \\
E_q[\log p(a_{\varepsilon_r})] &= \frac{1}{2}\log(A_{\varepsilon_r}^{-2}) - \log\Gamma(1/2) - \frac{3}{2}E_q[\log a_{\varepsilon_r}] - A_{\varepsilon_r}^{-2}E_q[1/a_{\varepsilon_r}] \\
E_q[\log p(a_k)] &= \frac{1}{2}\log(A_k^{-2}) - \log\Gamma(1/2) - \frac{3}{2}E_q[\log a_k] - A_k^{-2}E_q[1/a_k] \\
E_q[\log q(\beta,\mathbf{u})] &= \frac{-(p+mq)}{2}(1+\log(2\pi)) - \frac{1}{2}\log|\boldsymbol{\Sigma}_{q(\beta,\mathbf{u})}| \\
E_q[\log q(\boldsymbol{\Sigma}_R)] &= -\log C_{q,\nu+q+m-1} + \frac{\nu+q+m-1}{2}E_q[\log|B_{q(\Sigma_R)}|] - \frac{\nu+2q+m}{2}E_q[\log|\Sigma_R|] \\
&\quad - \frac{1}{2}E_q[\text{tr}(B_{q(\Sigma_R)}\Sigma_R^{-1})] \\
E_q[\log q(\sigma^2_{\varepsilon r})] &= \frac{1}{2}(\sum_{i=1}^m n_{ir}+1)E_q[\log B_{q(\sigma^2_{\varepsilon r})}] - \log\Gamma(1/2(\sum_{i=1}^m n_{ir}+1)) \\
&\quad - \left\{\frac{1}{2}(\sum_{i=1}^m n_{ir}+1)+1\right\}E_q[\log\sigma^2_{\varepsilon r}] - E_q\left[\frac{B_{q(\sigma^2_{\varepsilon r})}}{\sigma^2_{\varepsilon r}}\right] \\
E_q[\log q(a_{\varepsilon r})] &= E_q[\log B_{q(a_{\epsilon r})}] - \log\Gamma(1) - 2E_q[\log a_{\varepsilon_r}] - E_q\left[\frac{B_{q(a_{\varepsilon r})}}{a_{\varepsilon r}}\right] \\
E_q[\log q(a_k)] &= \frac{1}{2}(\nu+q)E_q[\log B_{q(a_k)}] - \log\Gamma(1/2(\nu+q)) - (1/2(\nu+q)+1)E_q[\log a_k] \\
&\quad - E_q\left[\frac{B_{q(a_k)}}{a_k}\right]
\end{aligned}
$$

In the above, $C_{d,A}$ is the normalising factor for the inverse-Wishart distribution given by

$$C_{d,A} = 2^{Ad/2}\pi^{d(d-1)/4}\prod_{i=1}^d\Gamma\left(\frac{A+1-i}{2}\right).$$

After evaluation of the expectations, and cancellation, we arrive at the expression for the lower bound on the marginal log-likelihood,

$$\begin{aligned}\log \underline{p}(\mathbf{y}, q) = & \frac{p+mq}{2} - n_G \log 2\pi - \log C_{q,\nu+q-1} + \log C_{q,\nu+q+m-1} \\ & - (R_c + q/2)\log\pi + q\log\Gamma(1/2(\nu+q)) - \frac{p}{2}\log(\sigma^2_\beta) + \frac{q(\nu+q+1)}{2}\log 2\nu \\ & + 1/2\log|\boldsymbol{\Sigma}_{q(\beta,\mathbf{u})}| + \mathbf{y}^T\mathbf{E}_2\mathbf{C}\mu_{q(\beta,\mathbf{u})} - \mathbf{1}^T\mathbf{E}_2\log\mathbf{y}! \\ & - \mathbf{1}^T\mathbf{E}_2\exp(\mathbf{C}\mu_{q(\beta,\mathbf{u})} + 1/2\text{diagonal}(\mathbf{C}\boldsymbol{\Sigma}_{q(\beta,\mathbf{u})}\mathbf{C}^T)) \\ & + \mathbf{y}^T\mathbf{E}_3\mathbf{C}\mu_{q(\beta,\mathbf{u})} - \mathbf{1}^T\mathbf{E}_3\tilde{\mathbf{B}}_0 - \frac{(\nu+q+m-1)}{2}\log|B_{q(\Sigma_R)}| - \frac{1}{2\sigma^2_\beta}(\mu^T_{q(\beta)}\mu_{q(\beta)} + \text{tr}(\Sigma_{q(\beta)})) \\ & + \sum_{r=1}^{R_c}\left\{\log\Gamma(1/2(\sum_{i=1}^m n_{ir}+1)) - \log A_{\varepsilon r - 1/2(\sum_{i=1}^m n_{ir}+1)\log B_{q(\sigma^2_{\varepsilon r})} - B_{q(a_{\varepsilon r})} + \mu_{q(1/a_{\varepsilon r})}\mu_{q(1/\sigma^2_{\varepsilon r})}}\right\} \\ & + \sum_{k=1}^{q}\left\{\nu\mathbf{M}_{q(\Sigma_R^{-1})_{kk}}\mu_{q(1/a_k)} - \log A_k - \frac{1}{2}(\nu+q)\log B_{q(a_k)}\right\}\end{aligned}$$

4. Additional simulation details

The final part of this supplement presents some accuracy results from the simulation study. The main manuscript presents the accuracy for the case where there are $m = 100 patients$. In the supplement, Figures 4 and 4 report the accuracy for the simulations with $m = 1000$ and $m = 10,000$ individuals respectively.

References

Harville, David A. (2008). *Matrix algebra from a statistician's perspective*. Springer, New York.

Lee, Cathy Yuen Yi and Wand, Matt P. (2016). Streamlined mean field variational bayes for longitudinal and multilevel data analysis. *Biometrical Journal* **58**(4), 868–895.

Nolan, Tui H and Wand, Matt P. (2017). Accurate logistic variational message passing: algebraic and numerical details. *Stat* **6**(1), 102–112.

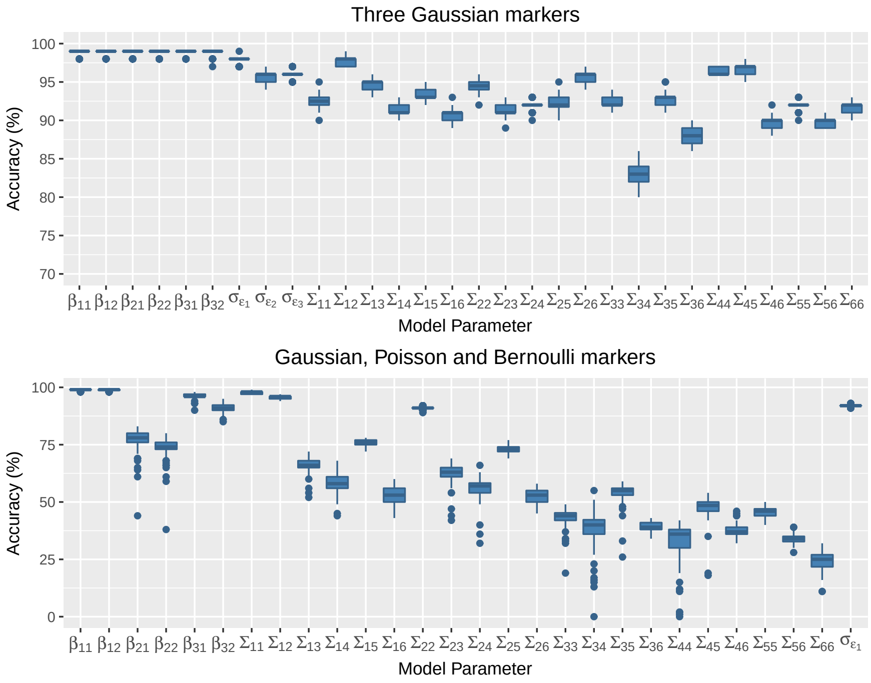

Fig. 1. Accuracy scores for mean field variational Bayes compared to MCMC for simulated datasets with 3 continuous longitudinal markers (top panel) and 3 types of markers (bottom panel) in the simulation with $m = 1000$ individuals

Rohde, David and Wand, Matt P. (2016). Semiparametric mean field variational bayes: General principles and numerical issues. *The Journal of Machine Learning Research* **17**(1), 5975–6021.

Wand, MP. (2002). Vector differential calculus in statistics. *The American Statistician* **56**(1), 55–62.

[*Received August 1, 2010; revised October 1, 2010; accepted for publication November 1, 2010*]

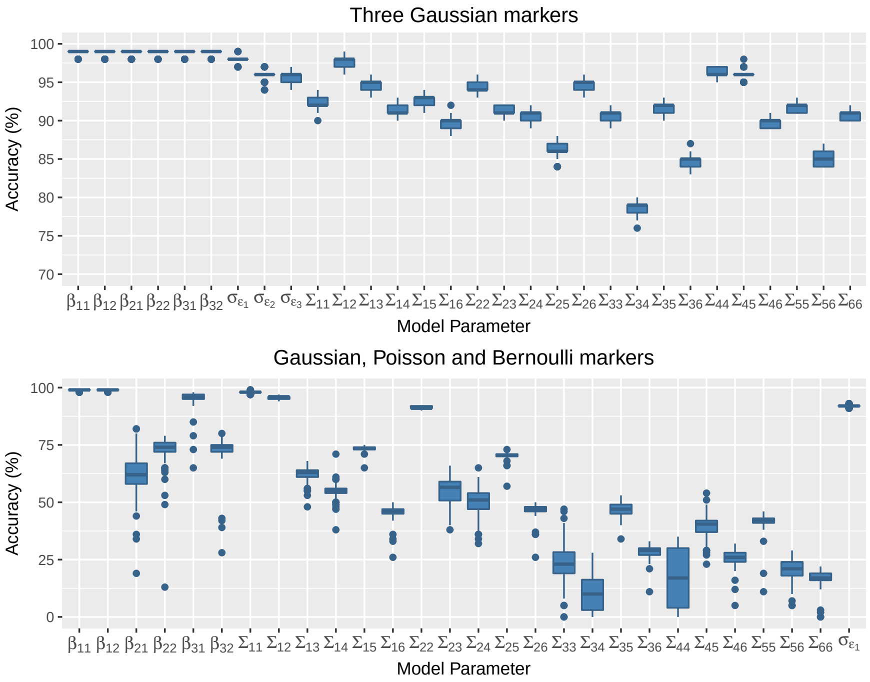

Fig. 2. Accuracy scores for mean field variational Bayes compared to MCMC for simulated datasets with 3 continuous longitudinal markers (top panel) and 3 types of markers (bottom panel) in the simulation with $m = 10000$ individuals

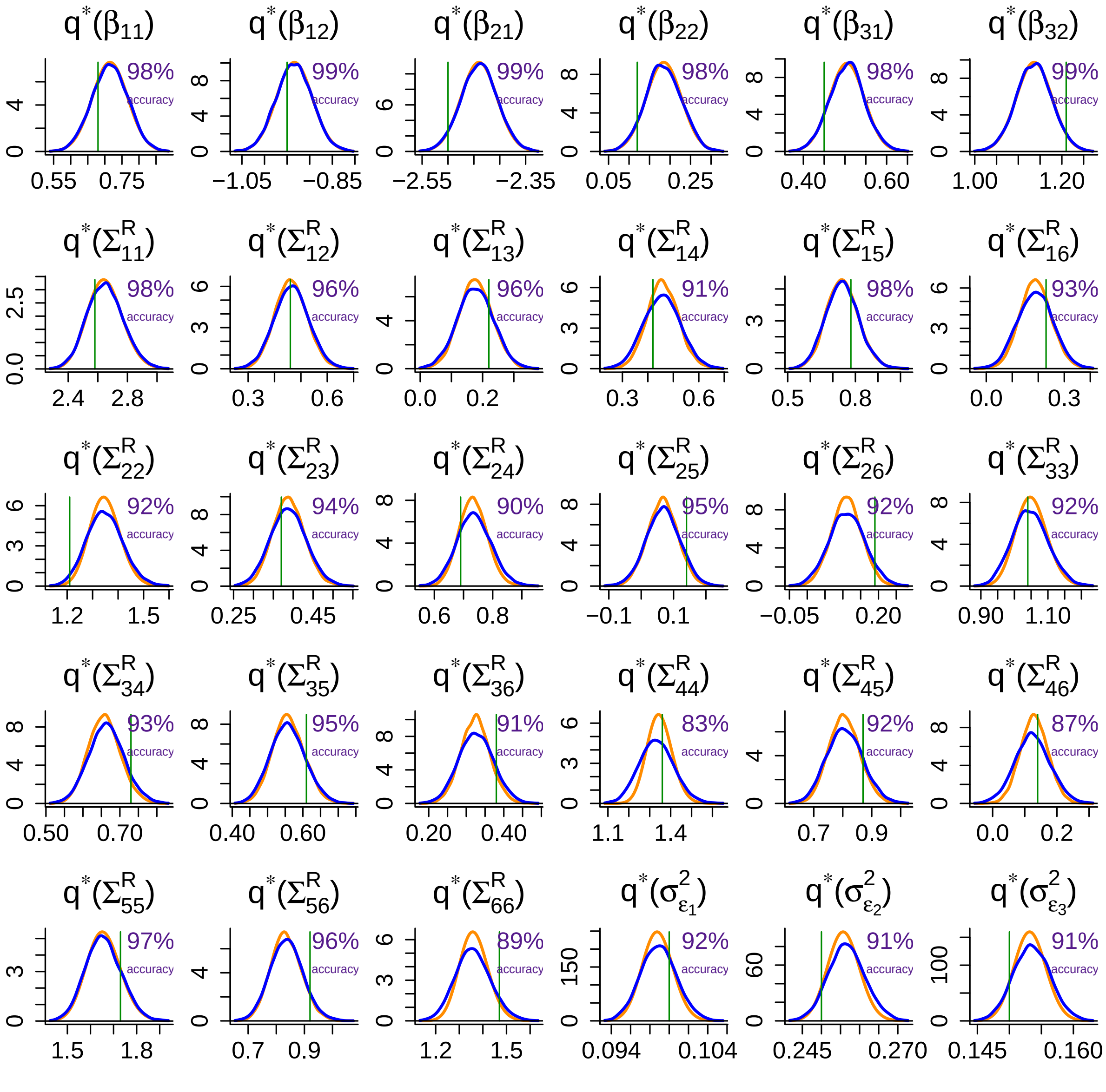

Fig. 3. Accuracy scores for mean field variational Bayes compared to MCMC for simulated datasets with 3 continuous longitudinal markers and $m = 1000$ individuals. The MCMC posteriors are shown by the blue densities, whilst the MFVB posterior densities are in orange. True values are shown by the green vertical line.

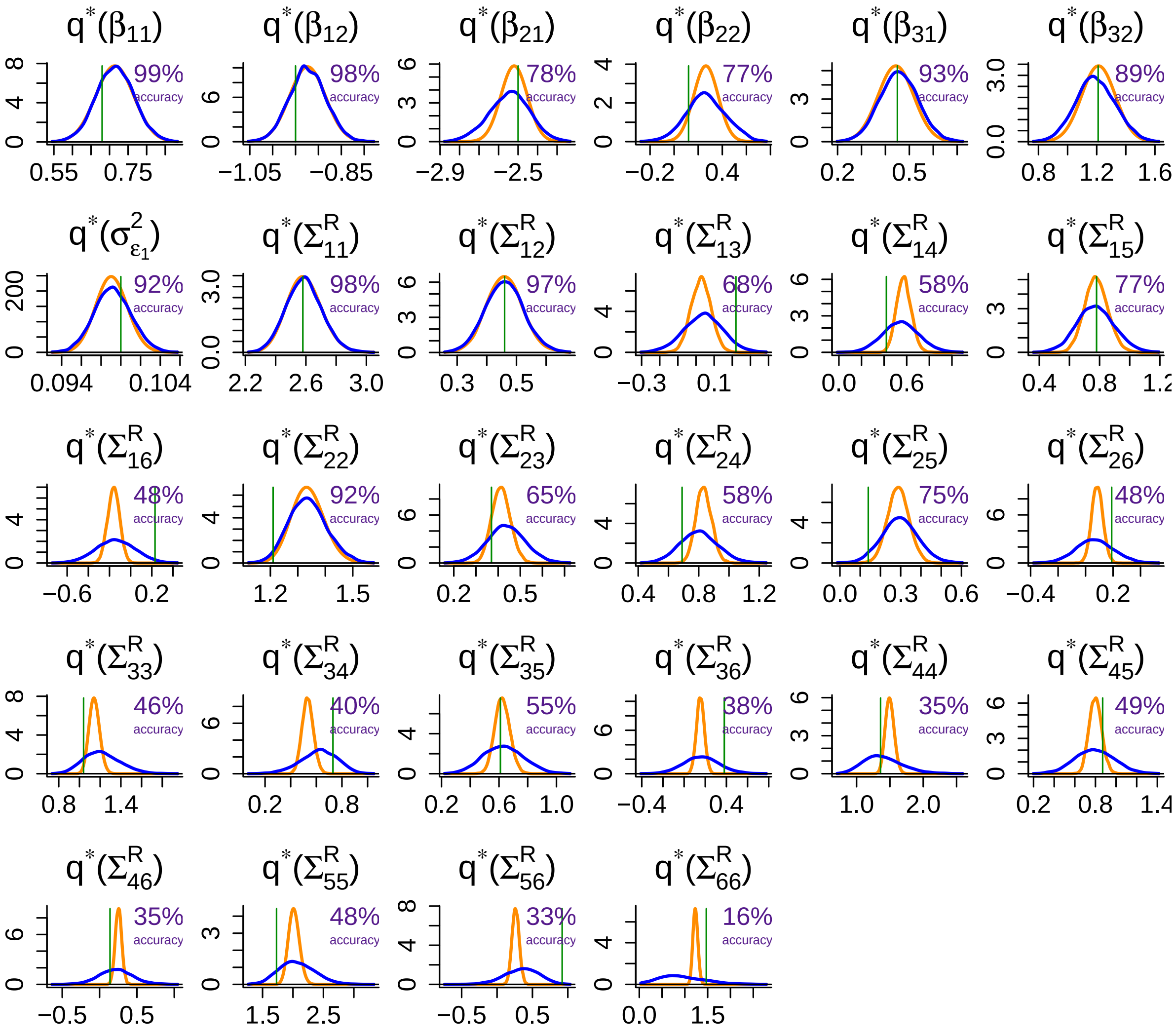

Fig. 4. Accuracy scores for mean field variational Bayes compared to MCMC for simulated datasets with one continuous, one binary and one Poisson longitudinal marker and $m = 1000$ individuals. The MCMC posteriors are shown by the blue densities, whilst the MFVB posterior densities are in orange. True values are shown by the green vertical line.

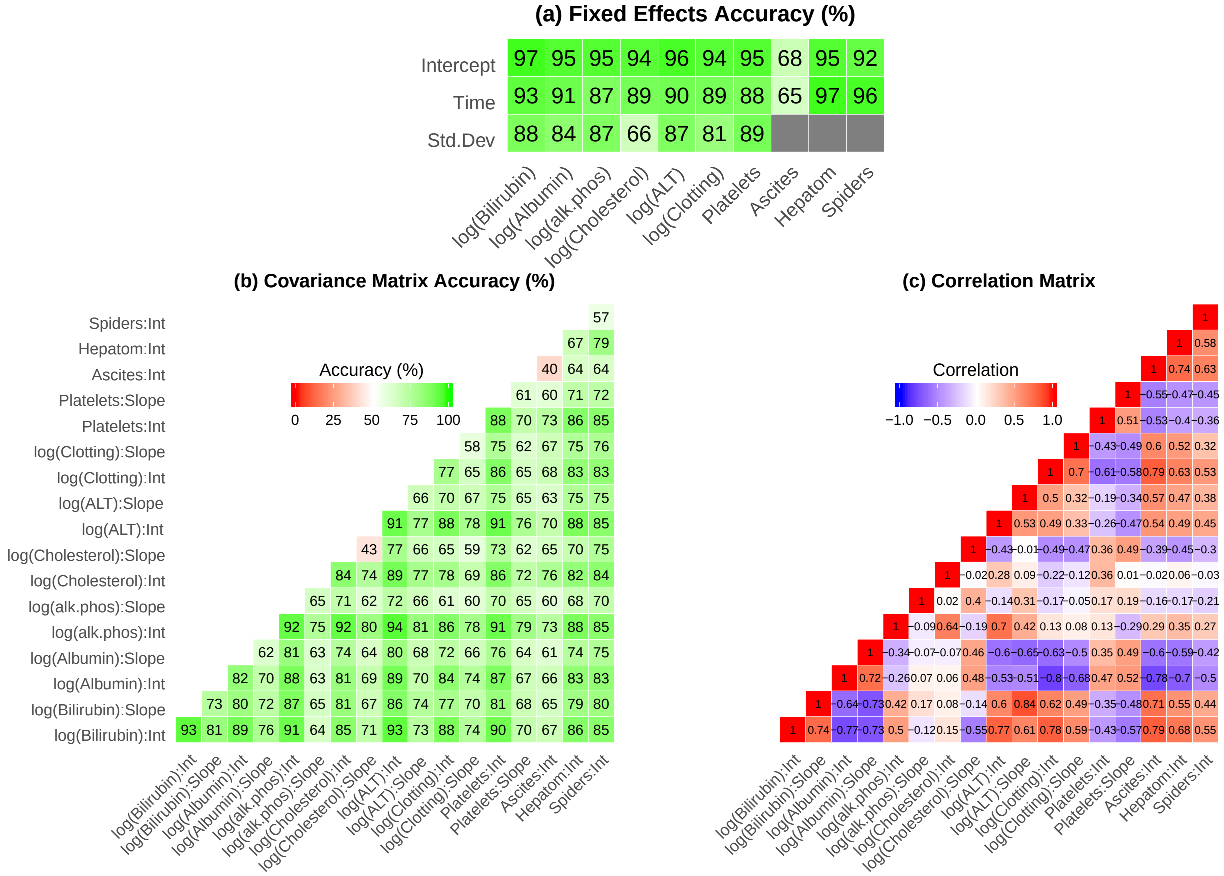

Fig. 5. Model results for a 10 marker multivariate generalised linear mixed model in the primary biliary cirrhosis data. Panel (a) shows the accuracy heat maps of the MFVB fixed effects estimates and residual standard deviations (compared to the MCMC estimates), (b) shows the accuracy of the MFVB random effects covariance matrix (compared to the MCMC estimates), and (c) shows the implied matrix of correlations between the 10 longitudinal markers calculated using MFVB.
